# Practical methods for incorporating summary time-to-event data into meta-analysis

**DOI:** 10.1186/1745-6215-8-16

**Published:** 2007-06-07

**Authors:** Jayne F Tierney, Lesley A Stewart, Davina Ghersi, Sarah Burdett, Matthew R Sydes

**Affiliations:** 1 Meta-analysis Group, MRC Clinical Trials Unit, London, UK; 2 Centre for Reviews and Dissemination, University of York, UK; 3 School of Public Health, NHMRC Clinical Trials Centre, Sydney, Australia; 4 Cancer Group, MRC Clinical Trials Unit, London, UK

## Abstract

**Background:**

In systematic reviews and meta-analyses, time-to-event outcomes are most appropriately analysed using hazard ratios (HRs). In the absence of individual patient data (IPD), methods are available to obtain HRs and/or associated statistics by carefully manipulating published or other summary data. Awareness and adoption of these methods is somewhat limited, perhaps because they are published in the statistical literature using statistical notation.

**Methods:**

This paper aims to 'translate' the methods for estimating a HR and associated statistics from published time-to-event-analyses into less statistical and more practical guidance and provide a corresponding, easy-to-use calculations spreadsheet, to facilitate the computational aspects.

**Results:**

A wider audience should be able to understand published time-to-event data in individual trial reports and use it more appropriately in meta-analysis. When faced with particular circumstances, readers can refer to the relevant sections of the paper. The spreadsheet can be used to assist them in carrying out the calculations.

**Conclusion:**

The methods cannot circumvent the potential biases associated with relying on published data for systematic reviews and meta-analysis. However, this practical guide should improve the quality of the analysis and subsequent interpretation of systematic reviews and meta-analyses that include time-to-event outcomes.

## Background

Time-to-event outcomes take account of whether an event takes place and also the time at which the event occurs, such that both the event and the timing of the event are important. For example, in cancer a cure may not be possible, but it is hoped that a new intervention will increase the duration of survival. Therefore, although the same or similar number of deaths may be observed, it is hoped that a new intervention will decrease the rate at which they take place. Other examples of outcomes where the timing of events may be vital in assessing the value of an intervention include: time free of seizures in epilepsy; time to conception in fertility treatment; time to resolution of symptoms of flu and time to fever in chickenpox.

Odds ratios (ORs) or relative risks (RRs) that measure only the number of events and take no account of when they occur are appropriate for measuring dichotomous outcomes, but less appropriate for analysing time-to-event outcomes. Using such dichotomous measures in a meta-analysis of time-to-event outcomes can pose additional problems. If the total number of events reported for each trial is used to calculate an OR or RR, this can involve combining trials reported at different stages of maturity, with variable follow up, resulting in an estimate that is both unreliable and difficult to interpret. Alternatively, ORs or RRs can be calculated at specific points in time making estimates comparable and easier to interpret, at least at those time-points. However, interpretation is difficult, particularly if individual trials do not contribute data at each time point. Furthermore, bias could arise if the time points are subjectively chosen by the systematic reviewer or selectively reported by the trialist at times of maximal or minimal difference between intervention groups.

Time-to-event outcomes are most appropriately analysed using hazard ratios (HRs), which take into account of the number and timing of events, and the time until last follow-up for each patient who has not experienced an event i.e. has been censored. HRs can be estimated by carefully manipulating published or other summary data [1,2], but currently such methods are under-used in meta-analyses. For example, Issue 3, 2006 of the Cochrane Library contained 43 cancer meta-analyses based on published data that included an analysis of survival and were not conducted by the current authors. Only sixteen of these estimated HRs and the remainder calculated ORs or RRs. This may reflect that the trials included in these meta-analyses did not report the necessary statistical information [3,4] to allow estimation of HRs. However, if there is sufficient data available to estimate an OR or RR, there is usually sufficient data to estimate a HR. Therefore, we suspect that use of the methods is limited because awareness is limited or because the statistical notation used to describe them may be difficult to follow for those with little formal statistical training. Furthermore, it is common for information on the effects of interventions to be presented in a number of different ways and it may not be clear which of the published methods is most appropriate.

Our aim in this paper is to provide step-by-step guidance on how to calculate a HR and the associated statistics for individual trials, according to the information presented in the trial report. To facilitate this we have translated the relevant equations (Appendix 1) from the previously reported statistical methods [1,2] into more descriptive versions, using familiar terms and explaining all arithmetic manipulations as simply as possible. We illustrate their use with data extracted from two cancer trial reports [5,6].

### Basic requirements for a meta-analysis based on hazard ratios

A meta-analysis of HRs, in common with meta-analyses of other effect measures, such as the RR or OR, usually involves a 2-stage process. In the first stage, a HR is estimated for each trial and in the second stage, these HRs are pooled in a meta-analysis. A fixed-effect meta-analysis of HRs, can use the method of Peto[7]:

pooled lnHR=[∑logrank Observed−Expected events(O−E)∑logrank Variance(V)]
 MathType@MTEF@5@5@+=feaafiart1ev1aaatCvAUfKttLearuWrP9MDH5MBPbIqV92AaeXatLxBI9gBaebbnrfifHhDYfgasaacH8akY=wiFfYdH8Gipec8Eeeu0xXdbba9frFj0=OqFfea0dXdd9vqai=hGuQ8kuc9pgc9s8qqaq=dirpe0xb9q8qiLsFr0=vr0=vr0dc8meaabaqaciaacaGaaeqabaqabeGadaaakeaacqqGWbaCcqqGVbWBcqqGVbWBcqqGSbaBcqqGLbqzcqqGKbazcqqGGaaicqqGSbaBcqqGUbGBcqqGibascqqGsbGucqGH9aqpdaWadaqaamaalaaabaWaaabqaeaaieGacqWFSbaBcqWFVbWBcqWFNbWzcqWGYbGCcqWGHbqycqWGUbGBcqWGRbWAcqqGGaaicqWGpbWtcqWGIbGycqWGZbWCcqWGLbqzcqWGYbGCcqWG2bGDcqWGLbqzcqWGKbazcqGHsislcqWGfbqrcqWG4baEcqWGWbaCcqWGLbqzcqWGJbWycqWG0baDcqWGLbqzcqWGKbazcqqGGaaicqWGLbqzcqWG2bGDcqWGLbqzcqWGUbGBcqWG0baDcqWGZbWCcqGGOaakcqWGpbWtcqGHsislcqWGfbqrcqGGPaqkaSqabeqaniabggHiLdaakeaadaaeabqaaiab=XgaSjab=9gaVjab=DgaNjabdkhaYjabdggaHjabd6gaUjabdUgaRjabbccaGiabdAfawjabdggaHjabdkhaYjabdMgaPjabdggaHjabd6gaUjabdogaJjabdwgaLjabcIcaOiabdAfawjabcMcaPaWcbeqab0GaeyyeIuoaaaaakiaawUfacaGLDbaaaaa@8878@

where ∑ is the "sum of" the respective values for each trial and "ln" is the natural logarithm (log). The *logrank Observed minus Expected events (O-E) *and the *logrank Variance (V) *are derived from the number of events and the individual times to event on the research arm of each trial. Alternatively, the inverse variance approach can be used [1]:

pooled lnHR=[∑log Hazard Ratio(lnHR)Variance of the lnHR(V∗)∑1Variance of the lnHR(V∗)]
 MathType@MTEF@5@5@+=feaafiart1ev1aaatCvAUfKttLearuWrP9MDH5MBPbIqV92AaeXatLxBI9gBaebbnrfifHhDYfgasaacH8akY=wiFfYdH8Gipec8Eeeu0xXdbba9frFj0=OqFfea0dXdd9vqai=hGuQ8kuc9pgc9s8qqaq=dirpe0xb9q8qiLsFr0=vr0=vr0dc8meaabaqaciaacaGaaeqabaqabeGadaaakeaacqqGWbaCcqqGVbWBcqqGVbWBcqqGSbaBcqqGLbqzcqqGKbazcqqGGaaicqqGSbaBcqqGUbGBcqqGibascqqGsbGucqGH9aqpdaWadaqaamaalaaabaWaaabqaeaadaWcaaqaaGqaciab=XgaSjab=9gaVjab=DgaNjabbccaGiabdIeaijabdggaHjabdQha6jabdggaHjabdkhaYjabdsgaKjabbccaGiabdkfasjabdggaHjabdsha0jabdMgaPjabd+gaVjabcIcaOiabbYgaSjabb6gaUjabbIeaijabbkfasjabcMcaPaqaaiabdAfawjabdggaHjabdkhaYjabdMgaPjabdggaHjabd6gaUjabdogaJjabdwgaLjabbccaGiabd+gaVjabdAgaMjabbccaGiabdsha0jabdIgaOjabdwgaLjabbccaGiabbYgaSjabb6gaUjabbIeaijabbkfasjabcIcaOiabdAfawnaaCaaaleqabaGaey4fIOcaaOGaeiykaKcaaaWcbeqab0GaeyyeIuoaaOqaamaaqaeabaWaaSaaaeaacqaIXaqmaeaacqWGwbGvcqWGHbqycqWGYbGCcqWGPbqAcqWGHbqycqWGUbGBcqWGJbWycqWGLbqzcqqGGaaicqWGVbWBcqWGMbGzcqqGGaaicqWG0baDcqWGObaAcqWGLbqzcqqGGaaicqqGSbaBcqqGUbGBcqqGibascqqGsbGucqGGOaakcqWGwbGvdaahaaWcbeqaaiabgEHiQaaakiabcMcaPaaaaSqabeqaniabggHiLdaaaaGccaGLBbGaayzxaaaaaa@97A4@

which uses the *Variance of the lnHR *(V*)and the *log Hazard Ratio (lnHR) for each trial*.

If the HR and V or lnHR and V* are presented in a trial report, they can be used directly in a fixed effect meta-analysis using (1) or (2) respectively. Similarly, if the coefficient of the treatment effect and the variance from a Cox model are provided, which correspond to the lnHR and V*, they too be used directly in a fixed effect meta-analysis using (2). These same statistics can be employed if a random effects meta-analysis [8] is required. Where they are not reported however, it is necessary to estimate the *O-E *and *V *or the lnHR and V* for each trial, in order to combine them in a meta-analysis.

### Generating the O-E, V, HR and lnHR from reported summary statistics

There are many ways to use the summary statistical data presented in trial reports to estimate the *O-E*, *V*, V*, HR and lnHR. Some methods use the reported information to directly calculate the HR or lnHR and V or V* and are described in Sections 1–2. However, it is more likely that a trial report will only provide sufficient information to estimate some or all of the HR, lnHR, *O-E*, *V *and V* by indirect methods that make certain assumptions, and these indirect methods are described in sections 3–9. For some of these methods, it is necessary to estimate the *V *and then derive V* and others the converse approach. Each is the reciprocal of the other:

Variance of the lnHR(V∗)=1logrank Variance(V)
 MathType@MTEF@5@5@+=feaafiart1ev1aaatCvAUfKttLearuWrP9MDH5MBPbIqV92AaeXatLxBI9gBaebbnrfifHhDYfgasaacH8akY=wiFfYdH8Gipec8Eeeu0xXdbba9frFj0=OqFfea0dXdd9vqai=hGuQ8kuc9pgc9s8qqaq=dirpe0xb9q8qiLsFr0=vr0=vr0dc8meaabaqaciaacaGaaeqabaqabeGadaaakeaacqWGwbGvcqWGHbqycqWGYbGCcqWGPbqAcqWGHbqycqWGUbGBcqWGJbWycqWGLbqzcqqGGaaicqWGVbWBcqWGMbGzcqqGGaaicqWG0baDcqWGObaAcqWGLbqzcqqGGaaicqqGSbaBcqqGUbGBcqqGibascqqGsbGucqGGOaakcqWGwbGvdaahaaWcbeqaaiabgEHiQaaakiabcMcaPiabg2da9maalaaabaGaeGymaedabaacbiGae8hBaWMae83Ba8Mae83zaCMaemOCaiNaemyyaeMaemOBa4Maem4AaSMaeeiiaaIaemOvayLaemyyaeMaemOCaiNaemyAaKMaemyyaeMaemOBa4Maem4yamMaemyzauMaeiikaGIaemOvayLaeiykaKcaaaaa@636D@

logrank Variance(V)=1Variance of the lnHR(V∗)
 MathType@MTEF@5@5@+=feaafiart1ev1aaatCvAUfKttLearuWrP9MDH5MBPbIqV92AaeXatLxBI9gBaebbnrfifHhDYfgasaacH8akY=wiFfYdH8Gipec8Eeeu0xXdbba9frFj0=OqFfea0dXdd9vqai=hGuQ8kuc9pgc9s8qqaq=dirpe0xb9q8qiLsFr0=vr0=vr0dc8meaabaqaciaacaGaaeqabaqabeGadaaakeaaieGacqWFSbaBcqWFVbWBcqWFNbWzcqWGYbGCcqWGHbqycqWGUbGBcqWGRbWAcqqGGaaicqWGwbGvcqWGHbqycqWGYbGCcqWGPbqAcqWGHbqycqWGUbGBcqWGJbWycqWGLbqzcqGGOaakcqWGwbGvcqGGPaqkcqGH9aqpdaWcaaqaaiabigdaXaqaaiabdAfawjabdggaHjabdkhaYjabdMgaPjabdggaHjabd6gaUjabdogaJjabdwgaLjabbccaGiabd+gaVjabdAgaMjabbccaGiabdsha0jabdIgaOjabdwgaLjabbccaGiabbYgaSjabb6gaUjabbIeaijabbkfasjabcIcaOiabdAfawnaaCaaaleqabaGaey4fIOcaaOGaeiykaKcaaaaa@636D@

*V *is used to denote the *logrank Variance *and V* to denote the *variance of the lnHR*.

If even these indirect methods cannot be applied, then it may be possible to generate the necessary statistics from published Kaplan-Meier curves (sections 10–11). For any set of trials, it is likely that a number of these methods will be required, and for any one trial, it may be possible to use more than one method.

### Extraction of summary statistics from trial reports

At the outset, it is worthwhile extracting all the necessary descriptive and statistical information for the outcome of interest for each trial [9], using a standard form (e.g. Table 1). The term "research" is used to denote the research intervention and "control" to denote the standard or control arm. Numbers have been rounded to two decimal places for presentation, but not for the underlying calculations. Rounding should in fact be avoided when making these calculations.

**Table 1:** Suggested data collection form completed with data extracted from the report of the example trial in bladder cancer [6]

**Trial Reference: *BA06***	**(Chemotherapy)**	**(No chemotherapy)**
Randomisation ratio (e.g. 1:1)	1	1
Patients randomised	491	485
Patients analysed	491	485
Observed events	229	256
Logrank expected events	Not reported	Not reported
Hazard ratio, confidence interval (& level e.g. 95%)	0.85, CI 0.71 to1.02 (95%)	
Logrank variance	Not reported	
Logrank observed minus-expected events	Not reported	
Hazard ratio and confidence interval (& level e.g. 95%) or standard error or variance from adjusted or unadjusted Cox	Not reported	
Test statistic, 2-sided p-value to 2 significant figures (& test used e.g. logrank, Mantel-Haenzsel or Cox)	Not reported, 0.075 (logrank)	
Advantage to research or control?	Research	
Actuarial or Kaplan Meier curves reported?	Yes, Kaplan Meier	
Numbers at risk reported	Yes	
Follow-up details	Min = 14 months, Max = 82 months (Estimated from recruitment of 69 months, 11/9 – 7/95 and median follow-up of 48 months)	

#### 1. Report presents O & E or hazard rates on research and control arm

If both the observed (*O*) and logrank expected events (*E*) on the research and control arm are presented in a trial report, then the HR can be calculated directly as the ratio of the hazard rates:

HR=[Observed events research/logrank Expected events researchObserved events control/logrank Expected events control]
 MathType@MTEF@5@5@+=feaafiart1ev1aaatCvAUfKttLearuWrP9MDH5MBPbIqV92AaeXatLxBI9gBaebbnrfifHhDYfgasaacH8akY=wiFfYdH8Gipec8Eeeu0xXdbba9frFj0=OqFfea0dXdd9vqai=hGuQ8kuc9pgc9s8qqaq=dirpe0xb9q8qiLsFr0=vr0=vr0dc8meaabaqaciaacaGaaeqabaqabeGadaaakeaacqqGibascqqGsbGucqGH9aqpdaWadaqaamaalaaabaGaem4ta8KaemOyaiMaem4CamNaemyzauMaemOCaiNaemODayNaemyzauMaemizaqMaeeiiaaIaemyzauMaemODayNaemyzauMaemOBa4MaemiDaqNaem4CamNaeeiiaaIaemOCaiNaemyzauMaem4CamNaemyzauMaemyyaeMaemOCaiNaem4yamMaemiAaGMaei4la8ccbiGae8hBaWMae83Ba8Mae83zaCMaemOCaiNaemyyaeMaemOBa4Maem4AaSMaeeiiaaIaemyrauKaemiEaGNaemiCaaNaemyzauMaem4yamMaemiDaqNaemyzauMaemizaqMaeeiiaaIaemyzauMaemODayNaemyzauMaemOBa4MaemiDaqNaem4CamNaeeiiaaIaemOCaiNaemyzauMaem4CamNaemyzauMaemyyaeMaemOCaiNaem4yamMaemiAaGgabaGaem4ta8KaemOyaiMaem4CamNaemyzauMaemOCaiNaemODayNaemyzauMaemizaqMaeeiiaaIaemyzauMaemODayNaemyzauMaemOBa4MaemiDaqNaem4CamNaeeiiaaIaem4yamMaem4Ba8MaemOBa4MaemiDaqNaemOCaiNaem4Ba8MaemiBaWMaei4la8Iae8hBaWMae83Ba8Mae83zaCMaemOCaiNaemyyaeMaemOBa4Maem4AaSMaeeiiaaIaemyrauKaemiEaGNaemiCaaNaemyzauMaem4yamMaemiDaqNaemyzauMaemizaqMaeeiiaaIaemyzauMaemODayNaemyzauMaemOBa4MaemiDaqNaem4CamNaeeiiaaIaem4yamMaem4Ba8MaemOBa4MaemiDaqNaemOCaiNaem4Ba8MaemiBaWgaaaGaay5waiaaw2faaaaa@C447@

The associated *V *can also be calculated directly:

V=1[(1/Expected events research)+(1/Expected events control)]
 MathType@MTEF@5@5@+=feaafiart1ev1aaatCvAUfKttLearuWrP9MDH5MBPbIqV92AaeXatLxBI9gBaebbnrfifHhDYfgasaacH8akY=wiFfYdH8Gipec8Eeeu0xXdbba9frFj0=OqFfea0dXdd9vqai=hGuQ8kuc9pgc9s8qqaq=dirpe0xb9q8qiLsFr0=vr0=vr0dc8meaabaqaciaacaGaaeqabaqabeGadaaakeaacqWGwbGvcqGH9aqpdaWcaaqaaiabigdaXaqaaiabcUfaBjabcIcaOiabigdaXiabc+caViabdweafjabdIha4jabdchaWjabdwgaLjabdogaJjabdsha0jabdwgaLjabdsgaKjabbccaGiabdwgaLjabdAha2jabdwgaLjabd6gaUjabdsha0jabdohaZjabbccaGiabdkhaYjabdwgaLjabdohaZjabdwgaLjabdggaHjabdkhaYjabdogaJjabdIgaOjabcMcaPiabgUcaRiabcIcaOiabigdaXiabc+caViabdweafjabdIha4jabdchaWjabdwgaLjabdogaJjabdsha0jabdwgaLjabdsgaKjabbccaGiabdwgaLjabdAha2jabdwgaLjabd6gaUjabdsha0jabdohaZjabbccaGiabdogaJjabd+gaVjabd6gaUjabdsha0jabdkhaYjabd+gaVjabdYgaSjabcMcaPiabc2faDbaaaaa@7830@

These statistics were included in our example report of an ovarian cancer trial [5]:

Observed events research = 34 Expected events research = 28.0

Observed events control = 24 Expected events control = 29.9

Using these data and equations (5) and (6), the HR and *V *can be calculated directly:

HR=34/28.024/29.9=1.51V=1[(1/28.0)+(1/29.9)]=14.46
 MathType@MTEF@5@5@+=feaafiart1ev1aaatCvAUfKttLearuWrP9MDH5MBPbIqV92AaeXatLxBI9gBaebbnrfifHhDYfgasaacH8akY=wiFfYdH8Gipec8Eeeu0xXdbba9frFj0=OqFfea0dXdd9vqai=hGuQ8kuc9pgc9s8qqaq=dirpe0xb9q8qiLsFr0=vr0=vr0dc8meaabaqaciaacaGaaeqabaqabeGadaaakeaafaqabeqacaaabaGaeeisaGKaeeOuaiLaeyypa0ZaaSaaaeaacqaIZaWmcqaI0aancqGGVaWlcqaIYaGmcqaI4aaocqGGUaGlcqaIWaamaeaacqaIYaGmcqaI0aancqGGVaWlcqaIYaGmcqaI5aqocqGGUaGlcqaI5aqoaaGaeyypa0JaeGymaeJaeiOla4IaeGynauJaeGymaedabaGaemOvayLaeyypa0ZaaSaaaeaacqaIXaqmaeaacqGGBbWwcqGGOaakcqaIXaqmcqGGVaWlcqaIYaGmcqaI4aaocqGGUaGlcqaIWaamcqGGPaqkcqGHRaWkcqGGOaakcqaIXaqmcqGGVaWlcqaIYaGmcqaI5aqocqGGUaGlcqaI5aqocqGGPaqkcqGGDbqxaaGaeyypa0JaeGymaeJaeGinaqJaeiOla4IaeGinaqJaeGOnaydaaaaa@5D0E@

The *O-E *is the number of observed events minus the logrank expected events on the research arm.

O - *E *= 34 - 28.0 = 6.00

If a hazard rate for each of the research and control arms is presented in a trial report they can replace the top and bottom of equation (5). Based on the example above, the hazard rate on the research arm of 1.21 and on control of 0.80 would be used to obtain a HR of 1.51. Such hazard rates cannot be used to calculate directly the associated *V*, which would need to be estimated using an indirect method (see below).

#### 2. Report presents O-E on research arm and logrank V

If a trial report presents the *O-E *events on the research arm and *V*, the HR can be calculated directly:

HR=exp⁡[Observed−Expected events research(O−E)Variance(V)]
 MathType@MTEF@5@5@+=feaafiart1ev1aaatCvAUfKttLearuWrP9MDH5MBPbIqV92AaeXatLxBI9gBaebbnrfifHhDYfgasaacH8akY=wiFfYdH8Gipec8Eeeu0xXdbba9frFj0=OqFfea0dXdd9vqai=hGuQ8kuc9pgc9s8qqaq=dirpe0xb9q8qiLsFr0=vr0=vr0dc8meaabaqaciaacaGaaeqabaqabeGadaaakeaacqqGibascqqGsbGucqGH9aqpcyGGLbqzcqGG4baEcqGGWbaCdaWadaqaamaalaaabaGaem4ta8KaemOyaiMaem4CamNaemyzauMaemOCaiNaemODayNaemyzauMaemizaqMaeyOeI0IaemyrauKaemiEaGNaemiCaaNaemyzauMaem4yamMaemiDaqNaemyzauMaemizaqMaeeiiaaIaemyzauMaemODayNaemyzauMaemOBa4MaemiDaqNaem4CamNaeeiiaaIaemOCaiNaemyzauMaem4CamNaemyzauMaemyyaeMaemOCaiNaem4yamMaemiAaGMaeiikaGIaem4ta8KaeyOeI0IaemyrauKaeiykaKcabaGaemOvayLaemyyaeMaemOCaiNaemyAaKMaemyyaeMaemOBa4Maem4yamMaemyzauMaeiikaGIaemOvayLaeiykaKcaaaGaay5waiaaw2faaaaa@73A9@

Note that "exp" represents the exponential or inverse of the natural log. HRs calculated using formula (7) will not differ markedly from the formal definition described previously (5), unless the event rate in a trial is low [1].

For illustration purposes, the data derived from the ovarian cancer trial report [5] are shown:

*O-E *= 6.00   *V *= 14.46

Using the calculated *O-E *and *V *in equation (7) gives a HR of 1.51:

HR=exp⁡[6.0014.46]=1.51
 MathType@MTEF@5@5@+=feaafiart1ev1aaatCvAUfKttLearuWrP9MDH5MBPbIqV92AaeXatLxBI9gBaebbnrfifHhDYfgasaacH8akY=wiFfYdH8Gipec8Eeeu0xXdbba9frFj0=OqFfea0dXdd9vqai=hGuQ8kuc9pgc9s8qqaq=dirpe0xb9q8qiLsFr0=vr0=vr0dc8meaabaqaciaacaGaaeqabaqabeGadaaakeaacqqGibascqqGsbGucqGH9aqpcyGGLbqzcqGG4baEcqGGWbaCdaWadaqaamaalaaabaGaeGOnayJaeiOla4IaeGimaaJaeGimaadabaGaeGymaeJaeGinaqJaeiOla4IaeGinaqJaeGOnaydaaaGaay5waiaaw2faaiabg2da9iabigdaXiabc6caUiabiwda1iabigdaXaaa@4360@

Note that equation (7) can be re-arranged by simple algebra thus:

V=[O−Eln⁡(HR)]
 MathType@MTEF@5@5@+=feaafiart1ev1aaatCvAUfKttLearuWrP9MDH5MBPbIqV92AaeXatLxBI9gBaebbnrfifHhDYfgasaacH8akY=wiFfYdH8Gipec8Eeeu0xXdbba9frFj0=OqFfea0dXdd9vqai=hGuQ8kuc9pgc9s8qqaq=dirpe0xb9q8qiLsFr0=vr0=vr0dc8meaabaqaciaacaGaaeqabaqabeGadaaakeaacqWGwbGvcqGH9aqpdaWadaqaamaalaaabaGaem4ta8KaeyOeI0IaemyraueabaGagiiBaWMaeiOBa4MaeiikaGIaeeisaGKaeeOuaiLaeiykaKcaaaGaay5waiaaw2faaaaa@3ACA@

*O-E *= ln(HR) × *V*

If the HR and *O-E *are reported, you can calculate *V. A*lternatively, if the HR and *V *are reported, you can calculate the *O-E*. Equations (8) and (9) are useful for some of the indirect methods presented later.

Equation (5) is the preferred estimate for the HR, although it will only differ markedly from (7) when the total number of events in a trial is small [1].

#### 3. Report presents HR and confidence intervals

Where the HR and its associated confidence interval (CI) are presented in a trial report, V* (variance of the ln(HR)) and subsequently, if necessary, *V*, can be estimated from the confidence interval (CI) provided the CI is given to two significant figures:

V∗=[ln⁡(upper CI)−ln⁡(lower CI)2×z score for upper CI boundary]2
 MathType@MTEF@5@5@+=feaafiart1ev1aaatCvAUfKttLearuWrP9MDH5MBPbIqV92AaeXatLxBI9gBaebbnrfifHhDYfgasaacH8akY=wiFfYdH8Gipec8Eeeu0xXdbba9frFj0=OqFfea0dXdd9vqai=hGuQ8kuc9pgc9s8qqaq=dirpe0xb9q8qiLsFr0=vr0=vr0dc8meaabaqaciaacaGaaeqabaqabeGadaaakeaacqWGwbGvdaahaaWcbeqaaiabgEHiQaaakiabg2da9maadmaabaWaaSaaaeaacyGGSbaBcqGGUbGBcqGGOaakcqqG1bqDcqqGWbaCcqqGWbaCcqqGLbqzcqqGYbGCcqqGGaaicqqGdbWqcqqGjbqscqGGPaqkcqGHsislcyGGSbaBcqGGUbGBcqGGOaakcqqGSbaBcqqGVbWBcqqG3bWDcqqGLbqzcqqGYbGCcqqGGaaicqqGdbWqcqqGjbqscqGGPaqkaeaacqaIYaGmcqGHxdaTcqqG6bGEcqqGGaaicqqGZbWCcqqGJbWycqqGVbWBcqqGYbGCcqqGLbqzcqqGGaaicqqGMbGzcqqGVbWBcqqGYbGCcqqGGaaicqqG1bqDcqqGWbaCcqqGWbaCcqqGLbqzcqqGYbGCcqqGGaaicqqGdbWqcqqGjbqscqqGGaaicqqGIbGycqqGVbWBcqqG1bqDcqqGUbGBcqqGKbazcqqGHbqycqqGYbGCcqqG5bqEaaaacaGLBbGaayzxaaWaaWbaaSqabeaacqaIYaGmaaaaaa@785B@

The top half of the equation uses the log of the upper and lower CI and the bottom half the z-score for the upper boundary of the confidence interval. In the usual situation of a 95% CI being presented, the corresponding z-score is 1.96. Thus, whenever a trial reports a HR and associated a 95% CI, this version of equation (10) can be used to calculate V*:

V∗=[ln⁡(upper 95%CI)−ln⁡(lower 95%CI)2×1.96]2
 MathType@MTEF@5@5@+=feaafiart1ev1aaatCvAUfKttLearuWrP9MDH5MBPbIqV92AaeXatLxBI9gBaebbnrfifHhDYfgasaacH8akY=wiFfYdH8Gipec8Eeeu0xXdbba9frFj0=OqFfea0dXdd9vqai=hGuQ8kuc9pgc9s8qqaq=dirpe0xb9q8qiLsFr0=vr0=vr0dc8meaabaqaciaacaGaaeqabaqabeGadaaakeaacqWGwbGvdaahaaWcbeqaaiabgEHiQaaakiabg2da9maadmaabaWaaSaaaeaacyGGSbaBcqGGUbGBcqGGOaakcqqG1bqDcqqGWbaCcqqGWbaCcqqGLbqzcqqGYbGCcqqGGaaicqaI5aqocqaI1aqncqGGLaqjcqqGdbWqcqqGjbqscqGGPaqkcqGHsislcyGGSbaBcqGGUbGBcqGGOaakcqqGSbaBcqqGVbWBcqqG3bWDcqqGLbqzcqqGYbGCcqqGGaaicqaI5aqocqaI1aqncqGGLaqjcqqGdbWqcqqGjbqscqGGPaqkaeaacqaIYaGmcqGHxdaTcqaIXaqmcqGGUaGlcqaI5aqocqaI2aGnaaaacaGLBbGaayzxaaWaaWbaaSqabeaacqaIYaGmaaaaaa@5D40@

For a 99% CI, the z-score is 2.58 and for a 90% CI the z-score is 1.64.

To demonstrate this and the rest of the indirect methods we use a report of a trial of chemotherapy versus no chemotherapy for bladder cancer [6]. The data extracted from the trial report data are shown in Table 1.

Inserting the 95% CI (0.71–1.02, Table 1) and the z-score of 1.96 into equation (10):

V∗=[ln⁡(1.02)−ln⁡(0.71)3.92]2=0.0085
 MathType@MTEF@5@5@+=feaafiart1ev1aaatCvAUfKttLearuWrP9MDH5MBPbIqV92AaeXatLxBI9gBaebbnrfifHhDYfgasaacH8akY=wiFfYdH8Gipec8Eeeu0xXdbba9frFj0=OqFfea0dXdd9vqai=hGuQ8kuc9pgc9s8qqaq=dirpe0xb9q8qiLsFr0=vr0=vr0dc8meaabaqaciaacaGaaeqabaqabeGadaaakeaacqWGwbGvdaahaaWcbeqaaiabgEHiQaaakiabg2da9maadmaabaWaaSaaaeaacyGGSbaBcqGGUbGBcqGGOaakcqaIXaqmcqGGUaGlcqaIWaamcqaIYaGmcqGGPaqkcqGHsislcyGGSbaBcqGGUbGBcqGGOaakcqaIWaamcqGGUaGlcqaI3aWncqaIXaqmcqGGPaqkaeaacqaIZaWmcqGGUaGlcqaI5aqocqaIYaGmaaaacaGLBbGaayzxaaWaaWbaaSqabeaacqaIYaGmaaGccqGH9aqpcqaIWaamcqGGUaGlcqaIWaamcqaIWaamcqaI4aaocqaI1aqnaaa@4EFB@

and using the estimated V* (without rounding) in equation (4):

V=10.0082=117.07
 MathType@MTEF@5@5@+=feaafiart1ev1aaatCvAUfKttLearuWrP9MDH5MBPbIqV92AaeXatLxBI9gBaebbnrfifHhDYfgasaacH8akY=wiFfYdH8Gipec8Eeeu0xXdbba9frFj0=OqFfea0dXdd9vqai=hGuQ8kuc9pgc9s8qqaq=dirpe0xb9q8qiLsFr0=vr0=vr0dc8meaabaqaciaacaGaaeqabaqabeGadaaakeaacqWGwbGvcqGH9aqpdaWcaaqaaiabigdaXaqaaiabicdaWiabc6caUiabicdaWiabicdaWiabiIda4iabikdaYaaacqGH9aqpcqaIXaqmcqaIXaqmcqaI3aWncqGGUaGlcqaIWaamcqaI3aWnaaa@3C35@

Gives an estimate of the logrank *V *of 117.07. Having both the reported HR of 0.85 and the estimated *V*, the *O-E *equation (9) can be used to obtain an O-E of -19.03

*O *- *E *= ln(0.85) × 117.07 = -19.03

Note that if a HR of an event on control versus the research arm is reported rather than vice versa, then a HR of the research arm versus control is obtained by taking the reciprocal of the HR i.e. 1/HR and associated CI.

#### 4. Report presents HR and events in each arm (and the randomisation ratio is 1:1)

Where a HR is reported, without the associated CI, but with the numbers of events on each arm, and the randomisation ratio is 1:1, a reasonable approximation of *V *may be obtained using equation (11):

V=Observed events research×Observed events controlTotal events
 MathType@MTEF@5@5@+=feaafiart1ev1aaatCvAUfKttLearuWrP9MDH5MBPbIqV92AaeXatLxBI9gBaebbnrfifHhDYfgasaacH8akY=wiFfYdH8Gipec8Eeeu0xXdbba9frFj0=OqFfea0dXdd9vqai=hGuQ8kuc9pgc9s8qqaq=dirpe0xb9q8qiLsFr0=vr0=vr0dc8meaabaqaciaacaGaaeqabaqabeGadaaakeaacqWGwbGvcqGH9aqpdaWcaaqaaiabd+eapjabdkgaIjabdohaZjabdwgaLjabdkhaYjabdAha2jabdwgaLjabdsgaKjabbccaGiabdwgaLjabdAha2jabdwgaLjabd6gaUjabdsha0jabdohaZjabbccaGiabdkhaYjabdwgaLjabdohaZjabdwgaLjabdggaHjabdkhaYjabdogaJjabdIgaOjabgEna0kabd+eapjabdkgaIjabdohaZjabdwgaLjabdkhaYjabdAha2jabdwgaLjabdsgaKjabbccaGiabdwgaLjabdAha2jabdwgaLjabd6gaUjabdsha0jabdohaZjabbccaGiabdogaJjabd+gaVjabd6gaUjabdsha0jabdkhaYjabd+gaVjabdYgaSbqaaiabdsfaujabd+gaVjabdsha0jabdggaHjabdYgaSjabbccaGiabdwgaLjabdAha2jabdwgaLjabd6gaUjabdsha0jabdohaZbaaaaa@7EE1@

Using the relevant data from the bladder cancer trial (Table 1), equation (11) and then equation (9):

V=229×256485=120.87O−E=ln⁡(0.85)×120.87=−19.64
 MathType@MTEF@5@5@+=feaafiart1ev1aaatCvAUfKttLearuWrP9MDH5MBPbIqV92AaeXatLxBI9gBaebbnrfifHhDYfgasaacH8akY=wiFfYdH8Gipec8Eeeu0xXdbba9frFj0=OqFfea0dXdd9vqai=hGuQ8kuc9pgc9s8qqaq=dirpe0xb9q8qiLsFr0=vr0=vr0dc8meaabaqaciaacaGaaeqabaqabeGadaaakeaafaqabeqacaaabaGaemOvayLaeyypa0ZaaSaaaeaacqaIYaGmcqaIYaGmcqaI5aqocqGHxdaTcqaIYaGmcqaI1aqncqaI2aGnaeaacqaI0aancqaI4aaocqaI1aqnaaGaeyypa0JaeGymaeJaeGOmaiJaeGimaaJaeiOla4IaeGioaGJaeG4naCdabaGaem4ta8KaeyOeI0IaemyrauKaeyypa0JagiiBaWMaeiOBa4MaeiikaGIaeGimaaJaeiOla4IaeGioaGJaeGynauJaeiykaKIaey41aqRaeGymaeJaeGOmaiJaeGimaaJaeiOla4IaeGioaGJaeG4naCJaeyypa0JaeyOeI0IaeGymaeJaeGyoaKJaeiOla4IaeGOnayJaeGinaqdaaaaa@5B6C@

Gives an estimate of 120.87 for *V *and -19.64 for the *O-E*.

#### 5. Report presents HR and total events (and the randomisation ratio is 1:1)

If only the total number of events is reported along with the HR, the variance can be approximated simply using the total number of events, provided again that the randomisation ratio is 1:1:

V=Total observed events4
 MathType@MTEF@5@5@+=feaafiart1ev1aaatCvAUfKttLearuWrP9MDH5MBPbIqV92AaeXatLxBI9gBaebbnrfifHhDYfgasaacH8akY=wiFfYdH8Gipec8Eeeu0xXdbba9frFj0=OqFfea0dXdd9vqai=hGuQ8kuc9pgc9s8qqaq=dirpe0xb9q8qiLsFr0=vr0=vr0dc8meaabaqaciaacaGaaeqabaqabeGadaaakeaacqWGwbGvcqGH9aqpdaWcaaqaaiabdsfaujabd+gaVjabdsha0jabdggaHjabdYgaSjabbccaGiabd+gaVjabdkgaIjabdohaZjabdwgaLjabdkhaYjabdAha2jabdwgaLjabdsgaKjabbccaGiabdwgaLjabdAha2jabdwgaLjabd6gaUjabdsha0jabdohaZbqaaiabisda0aaaaaa@4B8C@

where the total observed events is the sum of the observed events on the research and control arms.

Using the total number of events from the bladder cancer trial report (Table 1) gives an estimate 121.25 for *V*. Using this together with the reported HR and equation (9) gives a figure of -19.70 for the *O-E:*

V=4854=121.25O−E=ln⁡(0.85)×121.25=−19.70
 MathType@MTEF@5@5@+=feaafiart1ev1aaatCvAUfKttLearuWrP9MDH5MBPbIqV92AaeXatLxBI9gBaebbnrfifHhDYfgasaacH8akY=wiFfYdH8Gipec8Eeeu0xXdbba9frFj0=OqFfea0dXdd9vqai=hGuQ8kuc9pgc9s8qqaq=dirpe0xb9q8qiLsFr0=vr0=vr0dc8meaabaqaciaacaGaaeqabaqabeGadaaakeaafaqabeqacaaabaGaemOvayLaeyypa0ZaaSaaaeaacqaI0aancqaI4aaocqaI1aqnaeaacqaI0aanaaGaeyypa0JaeGymaeJaeGOmaiJaeGymaeJaeiOla4IaeGOmaiJaeGynaudabaGaem4ta8KaeyOeI0IaemyrauKaeyypa0JagiiBaWMaeiOBa4MaeiikaGIaeGimaaJaeiOla4IaeGioaGJaeGynauJaeiykaKIaey41aqRaeGymaeJaeGOmaiJaeGymaeJaeiOla4IaeGOmaiJaeGynauJaeyypa0JaeyOeI0IaeGymaeJaeGyoaKJaeiOla4IaeG4naCJaeGimaadaaaaa@5461@

This particular method of estimating *V *also provides a simple way of checking (approximately) the plausibility of estimates of *V *derived using other equations.

#### 6. Report presents HR, total events and the numbers randomised on each arm

If the randomisation ratio is not 1:1, methods 4 and 5 are not appropriate and one that accounts for the proportion of patients randomised to each arm is needed. If a report describes an analysis that is not based on all randomised patients; some patients being excluded subsequent to randomisation, then the HR and *V *should be based on the numbers analysed in the report rather than the numbers randomised, otherwise the precision of the estimate will be exaggerated:

V=Total observed events×Analysed research×Analysed control(Analysed research+Analysed control)2
 MathType@MTEF@5@5@+=feaafiart1ev1aaatCvAUfKttLearuWrP9MDH5MBPbIqV92AaeXatLxBI9gBaebbnrfifHhDYfgasaacH8akY=wiFfYdH8Gipec8Eeeu0xXdbba9frFj0=OqFfea0dXdd9vqai=hGuQ8kuc9pgc9s8qqaq=dirpe0xb9q8qiLsFr0=vr0=vr0dc8meaabaqaciaacaGaaeqabaqabeGadaaakeaacqWGwbGvcqGH9aqpdaWcaaqaaiabdsfaujabd+gaVjabdsha0jabdggaHjabdYgaSjabbccaGiabd+gaVjabdkgaIjabdohaZjabdwgaLjabdkhaYjabdAha2jabdwgaLjabdsgaKjabbccaGiabdwgaLjabdAha2jabdwgaLjabd6gaUjabdsha0jabdohaZjabgEna0kabdgeabjabd6gaUjabdggaHjabdYgaSjabdMha5jabdohaZjabdwgaLjabdsgaKjabbccaGiabdkhaYjabdwgaLjabdohaZjabdwgaLjabdggaHjabdkhaYjabdogaJjabdIgaOjabgEna0kabdgeabjabd6gaUjabdggaHjabdYgaSjabdMha5jabdohaZjabdwgaLjabdsgaKjabbccaGiabdogaJjabd+gaVjabd6gaUjabdsha0jabdkhaYjabd+gaVjabdYgaSbqaaiabcIcaOiabdgeabjabd6gaUjabdggaHjabdYgaSjabdMha5jabdohaZjabdwgaLjabdsgaKjabbccaGiabdkhaYjabdwgaLjabdohaZjabdwgaLjabdggaHjabdkhaYjabdogaJjabdIgaOjabgUcaRiabdgeabjabd6gaUjabdggaHjabdYgaSjabdMha5jabdohaZjabdwgaLjabdsgaKjabbccaGiabdogaJjabd+gaVjabd6gaUjabdsha0jabdkhaYjabd+gaVjabdYgaSjabcMcaPmaaCaaaleqabaGaeGOmaidaaaaaaaa@A981@

If more than one analysis is presented, for example, one based on eligible patients and one based on all randomised patients, it is preferable to use the analysis based on all randomised patients.

This method can also be used if the randomisation ratio is 1:1. In the bladder cancer trial report, all randomised patients were included in the analysis and so the number randomised in each arm equals the number analysed (Table 1). Equation (13) can be used to estimate *V *and equation (9) to estimate the *O-E*:

V=485×491×485(491+485)2=121.25O−E=ln⁡(0.85)×121.25=−19.70
 MathType@MTEF@5@5@+=feaafiart1ev1aaatCvAUfKttLearuWrP9MDH5MBPbIqV92AaeXatLxBI9gBaebbnrfifHhDYfgasaacH8akY=wiFfYdH8Gipec8Eeeu0xXdbba9frFj0=OqFfea0dXdd9vqai=hGuQ8kuc9pgc9s8qqaq=dirpe0xb9q8qiLsFr0=vr0=vr0dc8meaabaqaciaacaGaaeqabaqabeGadaaakeaafaqabeqacaaabaGaemOvayLaeyypa0ZaaSaaaeaacqaI0aancqaI4aaocqaI1aqncqGHxdaTcqaI0aancqaI5aqocqaIXaqmcqGHxdaTcqaI0aancqaI4aaocqaI1aqnaeaacqGGOaakcqaI0aancqaI5aqocqaIXaqmcqGHRaWkcqaI0aancqaI4aaocqaI1aqncqGGPaqkdaahaaWcbeqaaiabikdaYaaaaaGccqGH9aqpcqaIXaqmcqaIYaGmcqaIXaqmcqGGUaGlcqaIYaGmcqaI1aqnaeaacqWGpbWtcqGHsislcqWGfbqrcqGH9aqpcyGGSbaBcqGGUbGBcqGGOaakcqaIWaamcqGGUaGlcqaI4aaocqaI1aqncqGGPaqkcqGHxdaTcqaIXaqmcqaIYaGmcqaIXaqmcqGGUaGlcqaIYaGmcqaI1aqncqGH9aqpcqGHsislcqaIXaqmcqaI5aqocqGGUaGlcqaI3aWncqaIWaamaaaaaa@66FA@

For a trial that randomised patients according to a 1:1 ratio, but analysed unequal numbers of patients on each arm because, for example, patients were excluded differentially by arm, equation (13) is the preferred indirect method of estimating the variance.

#### 7. Report presents p-value and events in each arm (and the randomisation ratio is 1:1)

If only the logrank, Mantel Haenszel or even the Cox regression p-value, and numbers of events on each arm are reported and the randomisation ratio is 1:1, these data can be used to estimate the O-E using:

O−E=Observed events research×Observed events controlTotal observed events×(z score for p value÷2)
 MathType@MTEF@5@5@+=feaafiart1ev1aaatCvAUfKttLearuWrP9MDH5MBPbIqV92AaeXatLxBI9gBaebbnrfifHhDYfgasaacH8akY=wiFfYdH8Gipec8Eeeu0xXdbba9frFj0=OqFfea0dXdd9vqai=hGuQ8kuc9pgc9s8qqaq=dirpe0xb9q8qiLsFr0=vr0=vr0dc8meaabaqaciaacaGaaeqabaqabeGadaaakeaacqWGpbWtcqGHsislcqWGfbqrcqGH9aqpdaGcaaqaamaalaaabaGaem4ta8KaemOyaiMaem4CamNaemyzauMaemOCaiNaemODayNaemyzauMaemizaqMaeeiiaaIaemyzauMaemODayNaemyzauMaemOBa4MaemiDaqNaem4CamNaeeiiaaIaemOCaiNaemyzauMaem4CamNaemyzauMaemyyaeMaemOCaiNaem4yamMaemiAaGMaey41aqRaem4ta8KaemOyaiMaem4CamNaemyzauMaemOCaiNaemODayNaemyzauMaemizaqMaeeiiaaIaemyzauMaemODayNaemyzauMaemOBa4MaemiDaqNaem4CamNaeeiiaaIaem4yamMaem4Ba8MaemOBa4MaemiDaqNaemOCaiNaem4Ba8MaemiBaWgabaGaemivaqLaem4Ba8MaemiDaqNaemyyaeMaemiBaWMaeeiiaaIaem4Ba8MaemOyaiMaem4CamNaemyzauMaemOCaiNaemODayNaemyzauMaemizaqMaeeiiaaIaemyzauMaemODayNaemyzauMaemOBa4MaemiDaqNaem4CamhaaaWcbeaakiabgEna0kabcIcaOGqaciab=Pha6jabbccaGiabbohaZjabbogaJjabb+gaVjabbkhaYjabbwgaLjabbccaGiabbAgaMjabb+gaVjabbkhaYjabbccaGiabdchaWjabbccaGiabdAha2jabdggaHjabdYgaSjabdwha1jabdwgaLjabgEpa4kabikdaYiabcMcaPaaa@AB9F@

**Figure 1:**
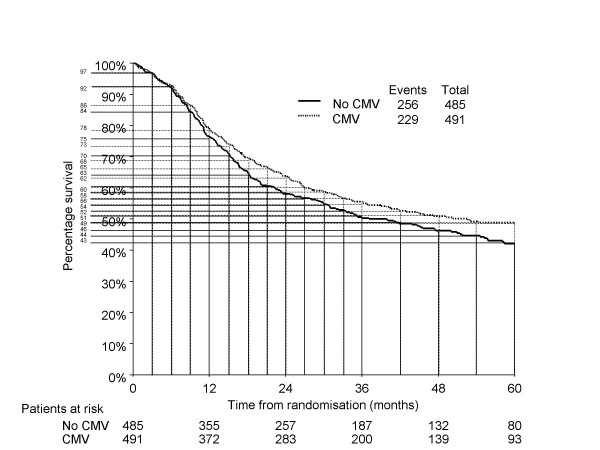
Bladder cancer trial Kaplan-Meier plot (modified with permission [6]), schematically divided into time intervals for data extraction into Table 2.

**Figure 2:**
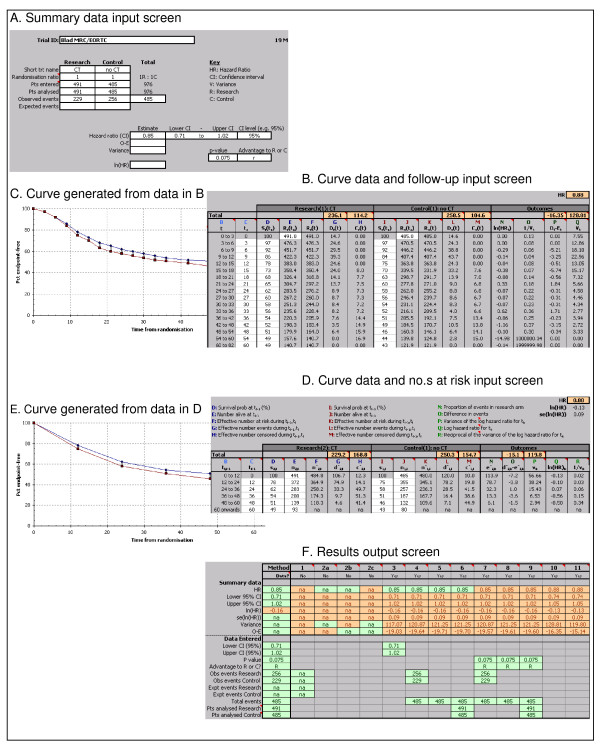
Data input screens (A, B and D), generated curves (C and E) and output screen (F) from the calculations spreadsheet.

For reliability, it is probably wise to use this method only when the exact p-value is given to at least 2 significant figures [1,2]. As well as the events on each arm and overall, a z-score for the 2-sided p-value divided by 2 is required. If a 1-sided p-value is reported it can be used directly to obtain the z-score. Such a z-score can be derived from either statistical tables or statistical or spreadsheet software (e.g. MS Excel).

A decision to assign a positive or negative value to *O-E *is needed and this depends on whether the direction of the effect is in favour of the research or control arm. This in turn will depend on whether the outcome is positive or negative. For a positive outcome, such as time to pregnancy, more pregnancies and/or a shorter the time to pregnancy on the research arm compared to the control arm, will indicate that the effect is in favour of the research arm. For a negative outcome, such as time to death, fewer deaths and/or a longer time to death on the research compared to the control arm will indicate that the effect is in favour of the research arm. If the results are not statistically significantly in favour of either the research or control arm or if the relative numbers of events on each arm are not provided, it is possible to look for other indicators of the direction of the results, such as the relative numbers of events on each arm, separation of Kaplan-Meier curves or textual descriptions of the results.

The logrank p-value of 0.075 gives a z-score of 1.78 and incorporating this with the number of events on each arm (Table 1) into equation (14):

O−E=229×256485×1.78=19.57
 MathType@MTEF@5@5@+=feaafiart1ev1aaatCvAUfKttLearuWrP9MDH5MBPbIqV92AaeXatLxBI9gBaebbnrfifHhDYfgasaacH8akY=wiFfYdH8Gipec8Eeeu0xXdbba9frFj0=OqFfea0dXdd9vqai=hGuQ8kuc9pgc9s8qqaq=dirpe0xb9q8qiLsFr0=vr0=vr0dc8meaabaqaciaacaGaaeqabaqabeGadaaakeaacqWGpbWtcqGHsislcqWGfbqrcqGH9aqpdaGcaaqaamaalaaabaGaeGOmaiJaeGOmaiJaeGyoaKJaey41aqRaeGOmaiJaeGynauJaeGOnaydabaGaeGinaqJaeGioaGJaeGynaudaaaWcbeaakiabgEna0kabigdaXiabc6caUiabiEda3iabiIda4iabg2da9iabigdaXiabiMda5iabc6caUiabiwda1iabiEda3aaa@478C@

gives an *O-E *of 19.57. It is clear from the report of the bladder cancer trial that survival favours the research treatment, with fewer deaths and a longer time to death in the research arm. Therefore, the *O-E *will be made negative (-19.57). Then, using equations (11) and (7):

V=229×256485=120.87HR=exp⁡[−19.57120.87]=0.85
 MathType@MTEF@5@5@+=feaafiart1ev1aaatCvAUfKttLearuWrP9MDH5MBPbIqV92AaeXatLxBI9gBaebbnrfifHhDYfgasaacH8akY=wiFfYdH8Gipec8Eeeu0xXdbba9frFj0=OqFfea0dXdd9vqai=hGuQ8kuc9pgc9s8qqaq=dirpe0xb9q8qiLsFr0=vr0=vr0dc8meaabaqaciaacaGaaeqabaqabeGadaaakeaafaqabeqacaaabaGaemOvayLaeyypa0ZaaSaaaeaacqaIYaGmcqaIYaGmcqaI5aqocqGHxdaTcqaIYaGmcqaI1aqncqaI2aGnaeaacqaI0aancqaI4aaocqaI1aqnaaGaeyypa0JaeGymaeJaeGOmaiJaeGimaaJaeiOla4IaeGioaGJaeG4naCdabaGaeeisaGKaeeOuaiLaeyypa0JagiyzauMaeiiEaGNaeiiCaa3aamWaaeaadaWcaaqaaiabgkHiTiabigdaXiabiMda5iabc6caUiabiwda1iabiEda3aqaaiabigdaXiabikdaYiabicdaWiabc6caUiabiIda4iabiEda3aaaaiaawUfacaGLDbaacqGH9aqpcqaIWaamcqGGUaGlcqaI4aaocqaI1aqnaaaaaa@5A32@

*V *is estimated as 120.87 and the HR as 0.85.

#### 8. Report presents p-value and total events (and the randomisation ratio is 1:1)

A similar equation to (14) can be used if just the p-value and the total number of events are reported, provided the randomisation ratio (or the ratio of patients analysed) is 1:1:

O−E=1/2×Total observed events×(z score for p value÷2)
 MathType@MTEF@5@5@+=feaafiart1ev1aaatCvAUfKttLearuWrP9MDH5MBPbIqV92AaeXatLxBI9gBaebbnrfifHhDYfgasaacH8akY=wiFfYdH8Gipec8Eeeu0xXdbba9frFj0=OqFfea0dXdd9vqai=hGuQ8kuc9pgc9s8qqaq=dirpe0xb9q8qiLsFr0=vr0=vr0dc8meaabaqaciaacaGaaeqabaqabeGadaaakeaacqWGpbWtcqGHsislcqWGfbqrcqGH9aqpcqaIXaqmcqGGVaWlcqaIYaGmcqGHxdaTdaGcaaqaaiabdsfaujabd+gaVjabdsha0jabdggaHjabdYgaSjabbccaGiabd+gaVjabdkgaIjabdohaZjabdwgaLjabdkhaYjabdAha2jabdwgaLjabdsgaKjabbccaGiabdwgaLjabdAha2jabdwgaLjabd6gaUjabdsha0jabdohaZbWcbeaakiabgEna0kabcIcaOiabbQha6jabbccaGiabbohaZjabbogaJjabb+gaVjabbkhaYjabbwgaLjabbccaGiabbAgaMjabb+gaVjabbkhaYjabbccaGiabdchaWjabbccaGiabdAha2jabdggaHjabdYgaSjabdwha1jabdwgaLjabgEpa4kabikdaYiabcMcaPaaa@7057@

Using equation (15):

O−E=1/2×485×1.78=19.60
 MathType@MTEF@5@5@+=feaafiart1ev1aaatCvAUfKttLearuWrP9MDH5MBPbIqV92AaeXatLxBI9gBaebbnrfifHhDYfgasaacH8akY=wiFfYdH8Gipec8Eeeu0xXdbba9frFj0=OqFfea0dXdd9vqai=hGuQ8kuc9pgc9s8qqaq=dirpe0xb9q8qiLsFr0=vr0=vr0dc8meaabaqaciaacaGaaeqabaqabeGadaaakeaacqWGpbWtcqGHsislcqWGfbqrcqGH9aqpcqaIXaqmcqGGVaWlcqaIYaGmcqGHxdaTdaGcaaqaaiabisda0iabiIda4iabiwda1aWcbeaakiabgEna0kabigdaXiabc6caUiabiEda3iabiIda4iabg2da9iabigdaXiabiMda5iabc6caUiabiAda2iabicdaWaaa@4470@

As before, a sign needs to be applied based on the direction of the results, giving -19.60. Then using (12) and (8):

V=4854=121.25HR=exp⁡[−19.60121.25]=0.85
 MathType@MTEF@5@5@+=feaafiart1ev1aaatCvAUfKttLearuWrP9MDH5MBPbIqV92AaeXatLxBI9gBaebbnrfifHhDYfgasaacH8akY=wiFfYdH8Gipec8Eeeu0xXdbba9frFj0=OqFfea0dXdd9vqai=hGuQ8kuc9pgc9s8qqaq=dirpe0xb9q8qiLsFr0=vr0=vr0dc8meaabaqaciaacaGaaeqabaqabeGadaaakeaafaqabeqacaaabaGaemOvayLaeyypa0ZaaSaaaeaacqaI0aancqaI4aaocqaI1aqnaeaacqaI0aanaaGaeyypa0JaeGymaeJaeGOmaiJaeGymaeJaeiOla4IaeGOmaiJaeGynaudabaGaeeisaGKaeeOuaiLaeyypa0JagiyzauMaeiiEaGNaeiiCaa3aamWaaeaadaWcaaqaaiabgkHiTiabigdaXiabiMda5iabc6caUiabiAda2iabicdaWaqaaiabigdaXiabikdaYiabigdaXiabc6caUiabikdaYiabiwda1aaaaiaawUfacaGLDbaacqGH9aqpcqaIWaamcqGGUaGlcqaI4aaocqaI1aqnaaaaaa@5321@

give estimates of 121.25 for *V *and 0.85 for the HR.

#### 9. Report presents p-value, total events and numbers randomised to each arm

Where the report presents the p-value, the total events and the numbers randomised on each arm, another equation similar to (14) allows estimation of the *O-E *for trials where the randomisation (or analysis) ratio is not 1:1:

O−E=(Total observed events×Analysed research×Analysed control)(Analysed research+Analysed control)×(z score for p value÷2)
 MathType@MTEF@5@5@+=feaafiart1ev1aaatCvAUfKttLearuWrP9MDH5MBPbIqV92AaeXatLxBI9gBaebbnrfifHhDYfgasaacH8akY=wiFfYdH8Gipec8Eeeu0xXdbba9frFj0=OqFfea0dXdd9vqai=hGuQ8kuc9pgc9s8qqaq=dirpe0xb9q8qiLsFr0=vr0=vr0dc8meaabaqaciaacaGaaeqabaqabeGadaaakeaacqWGpbWtcqGHsislcqWGfbqrcqGH9aqpdaWcaaqaamaakaaabaGaeiikaGIaemivaqLaem4Ba8MaemiDaqNaemyyaeMaemiBaWMaeeiiaaIaem4Ba8MaemOyaiMaem4CamNaemyzauMaemOCaiNaemODayNaemyzauMaemizaqMaeeiiaaIaemyzauMaemODayNaemyzauMaemOBa4MaemiDaqNaem4CamNaey41aqRaemyqaeKaemOBa4MaemyyaeMaemiBaWMaemyEaKNaem4CamNaemyzauMaemizaqMaeeiiaaIaemOCaiNaemyzauMaem4CamNaemyzauMaemyyaeMaemOCaiNaem4yamMaemiAaGMaey41aqRaemyqaeKaemOBa4MaemyyaeMaemiBaWMaemyEaKNaem4CamNaemyzauMaemizaqMaeeiiaaIaem4yamMaem4Ba8MaemOBa4MaemiDaqNaemOCaiNaem4Ba8MaemiBaWMaeiykaKcaleqaaaGcbaGaeiikaGIaemyqaeKaemOBa4MaemyyaeMaemiBaWMaemyEaKNaem4CamNaemyzauMaemizaqMaeeiiaaIaemOCaiNaemyzauMaem4CamNaemyzauMaemyyaeMaemOCaiNaem4yamMaemiAaGMaey4kaSIaemyqaeKaemOBa4MaemyyaeMaemiBaWMaemyEaKNaem4CamNaemyzauMaemizaqMaeeiiaaIaem4yamMaem4Ba8MaemOBa4MaemiDaqNaemOCaiNaem4Ba8MaemiBaWMaeiykaKcaaiabgEna0kabcIcaOiabbQha6jabbccaGiabbohaZjabbogaJjabb+gaVjabbkhaYjabbwgaLjabbccaGiabbAgaMjabb+gaVjabbkhaYjabbccaGiabdchaWjabbccaGiabdAha2jabdggaHjabdYgaSjabdwha1jabdwgaLjabgEpa4kabikdaYiabcMcaPaaa@CB06@

Using (16):

O−E=(485×491×485)(491+485)×1.78=19.60
 MathType@MTEF@5@5@+=feaafiart1ev1aaatCvAUfKttLearuWrP9MDH5MBPbIqV92AaeXatLxBI9gBaebbnrfifHhDYfgasaacH8akY=wiFfYdH8Gipec8Eeeu0xXdbba9frFj0=OqFfea0dXdd9vqai=hGuQ8kuc9pgc9s8qqaq=dirpe0xb9q8qiLsFr0=vr0=vr0dc8meaabaqaciaacaGaaeqabaqabeGadaaakeaacqWGpbWtcqGHsislcqWGfbqrcqGH9aqpdaWcaaqaamaakaaabaGaeiikaGIaeGinaqJaeGioaGJaeGynauJaey41aqRaeGinaqJaeGyoaKJaeGymaeJaey41aqRaeGinaqJaeGioaGJaeGynauJaeiykaKcaleqaaaGcbaGaeiikaGIaeGinaqJaeGyoaKJaeGymaeJaey4kaSIaeGinaqJaeGioaGJaeGynauJaeiykaKcaaiabgEna0kabigdaXiabc6caUiabiEda3iabiIda4iabg2da9iabigdaXiabiMda5iabc6caUiabiAda2iabicdaWaaa@53B9@

Applying a negative sign on the basis of the direction of the results (-19.60) and equations (13) and (8):

V=485×491×485(491+485)2=121.25HR=exp⁡[−19.60121.25]=0.85
 MathType@MTEF@5@5@+=feaafiart1ev1aaatCvAUfKttLearuWrP9MDH5MBPbIqV92AaeXatLxBI9gBaebbnrfifHhDYfgasaacH8akY=wiFfYdH8Gipec8Eeeu0xXdbba9frFj0=OqFfea0dXdd9vqai=hGuQ8kuc9pgc9s8qqaq=dirpe0xb9q8qiLsFr0=vr0=vr0dc8meaabaqaciaacaGaaeqabaqabeGadaaakeaafaqabeqacaaabaGaemOvayLaeyypa0ZaaSaaaeaacqaI0aancqaI4aaocqaI1aqncqGHxdaTcqaI0aancqaI5aqocqaIXaqmcqGHxdaTcqaI0aancqaI4aaocqaI1aqnaeaacqGGOaakcqaI0aancqaI5aqocqaIXaqmcqGHRaWkcqaI0aancqaI4aaocqaI1aqncqGGPaqkdaahaaWcbeqaaiabikdaYaaaaaGccqGH9aqpcqaIXaqmcqaIYaGmcqaIXaqmcqGGUaGlcqaIYaGmcqaI1aqnaeaacqqGibascqqGsbGucqGH9aqpcyGGLbqzcqGG4baEcqGGWbaCdaWadaqaamaalaaabaGaeyOeI0IaeGymaeJaeGyoaKJaeiOla4IaeGOnayJaeGimaadabaGaeGymaeJaeGOmaiJaeGymaeJaeiOla4IaeGOmaiJaeGynaudaaaGaay5waiaaw2faaiabg2da9iabicdaWiabc6caUiabiIda4iabiwda1aaaaaa@65BA@

Provides an estimate of 121.25 for the *V *and 0.85 for the HR.

### Generating the O-E, V, HR and lnHR from published Kaplan-Meier curves

Some time-to-event analyses are presented solely in the form of Kaplan-Meier curves [1,10]. It is possible to estimate the HR, lnHR, *O-E *and *V *from a number of time intervals from such curves and pool across these time intervals within a trial to estimate a HR or lnHR that represents the whole curve (section 10–11). Alongside, the reported minimum and maximum follow-up times or the reported numbers at risk can be used, to estimate the amount of censoring in a trial. Otherwise, the estimate of effect would be based on too many patients and so be erroneously precise. If a trial report does not present either the numbers at risk or the actual minimum and maximum follow-up, then it may be possible to estimate the level of follow-up from other information provided (Appendix 2).

### Extraction of curve data from trial reports

A sufficiently large, clear copy of the curve needs to be divided up into a number of time intervals, which give a good representation of event rates over time, whilst limiting the number of events within any time interval. Parmar *et al*. [1], suggest that, as far as possible, the event rate within a time interval should be no more than 20% of those at the start of the time interval. If the curve starts to level off, then few (or no) events are taking place and there is little value in extracting data from this area of a curve. Also, the final interval should not extend beyond the actual or estimated maximum follow-up.

For example, in a trial of metastatic breast cancer, many events (deaths) will occur in the first 3 months, so the curve would need to be split into smaller intervals at the beginning then gradually larger time intervals (e.g. monthly for the first 12 months, 3-monthly to 24 months and then 6-monthly thereafter). However, the curve from the bladder cancer trial (Figure 1) shows an event (death) rate that is quite high in the earlier parts of the curve, but is subsequently fairly steady. Therefore, the curve was divided into 3-monthly intervals for the first 3 years and 6-monthly intervals thereafter (Figure 1). The percentage survival for each arm at the start of each time interval, for each arm, was then extracted into Table 2.

**Table 2:** Example data extraction form with data extracted from bladder cancer Kaplan-Meier plot in Figure 1.

**Time at start of interval (months)**	**% Event-free on research**	**% Event-free on control**	**Reported numbers at risk on research**	**Reported numbers at risk on control**
0	100	100	491	485
3	97	97	-	-
6	92	92	-	-
9	86	84	-	-
12	78	75	372	355
15	73	70	-	-
18	68	63	-	-
21	65	60	-	-
24	62	58	283	257
27	60	56	-	-
30	58	54	-	-
33	56	52	-	-
36	54	51	200	187
42	52	49	-	-
48	51	46	139	132
54	49	44	-	-
60	49	43	93	80

### 10. Report presents Kaplan-Meier curve and information on follow-up

For each time interval and for each arm a number of iterative calculations are required. It is necessary to estimate the number of patients who were: 1) event-free at the start of the interval, 2) censored during the interval and 3) at risk during the interval. Also, 4) the number of events during each interval needs to be estimated. Together these items are used to: 5) estimate the *O-E*, *V *and HR for each time interval. Finally, 6) the *O-E*, *V *and HR for the whole curve are derived from combining the estimates across time interval.

The numbers of patients at risk at the start of the first time interval is simply the total number analysed on each arm, making step 1 redundant for the first time interval of any curve. Therefore, in the bladder cancer trial, at the start of the 0–3 month time period, there are 491 and 485 patients at risk on the research and control arms, respectively (Table 2).

Based on the median follow-up of 48 months and accrual period of 69 months (Table 1), the minimum follow-up is estimated (Appendix 2) to be 14 months for this trial, and so all patients have complete follow-up and no patients are censored in the 0–3, 3–6, 6–9 and 9–12 month intervals. Therefore, for these time intervals, estimating the number of patients censored (step 2) is not relevant. Beyond 14 months patients are censored and this must be taken into account. Going through the steps 1, 3, 4 and 5 for the prior time intervals, the following were estimated for the 12–15 month time interval:

Event-free at start of prior time interval (12–15 month), research = 382.98

Event-free at start of prior time interval (12–15 month), control = 363.75

Events in prior time interval (12–15 month), research = 24.55

Events in prior time interval (12–15 month), control = 24.25

Censored in prior time interval (12–15 month), research = 0.00

Censored in prior time interval (12–15 month), control = 0.00

Note that these estimated values differ somewhat from the actual reported numbers at risk at 12 months (Table 2), but they can be used to illustrate all the steps of the method, in the presence of censoring, for the 15–18 month interval:

#### Step 1. Numbers event-free at the start of the current interval

This is in fact the number of patients that were event-free at the end of the prior time interval:

*Event free at start of current interval *= *Event free at start of prior interval *- *Events in prior interval *- *Censored during prior interval*

Using the data from 12–15 month time interval, the numbers of patients event-free in the current 15–18 month time interval are estimated:

*Event free at start (15–18 month), research *= 382.98 - 24.55 - 0 = 358.43

*Event free at start (15–18 month), control *= 363.75 - 24.25 - 0 = 339.5

#### Step 2. Numbers censored during the current interval

Assuming that censoring is non-informative and that patients are censored at a constant rate within a given time interval, a simple method can be used to estimate numbers censored [1]:

At risk during current interval×(12×(End of time interval−Start of time interval)(Maximum follow up−Start of time interval))
 MathType@MTEF@5@5@+=feaafiart1ev1aaatCvAUfKttLearuWrP9MDH5MBPbIqV92AaeXatLxBI9gBaebbnrfifHhDYfgasaacH8akY=wiFfYdH8Gipec8Eeeu0xXdbba9frFj0=OqFfea0dXdd9vqai=hGuQ8kuc9pgc9s8qqaq=dirpe0xb9q8qiLsFr0=vr0=vr0dc8meaabaqaciaacaGaaeqabaqabeGadaaakeaacqWGbbqqcqWG0baDcqqGGaaicqWGYbGCcqWGPbqAcqWGZbWCcqWGRbWAcqqGGaaicqWGKbazcqWG1bqDcqWGYbGCcqWGPbqAcqWGUbGBcqWGNbWzcqqGGaaicqWGJbWycqWG1bqDcqWGYbGCcqWGYbGCcqWGLbqzcqWGUbGBcqWG0baDcqqGGaaiieGacqWFPbqAcqWFUbGBcqWF0baDcqWGLbqzcqWGYbGCcqWG2bGDcqWGHbqycqWGSbaBcqGHxdaTdaqadaqaamaalaaabaGaeGymaedabaGaeGOmaidaaiabgEna0oaalaaabaGaeiikaGIaemyrauKaemOBa4MaemizaqMaeeiiaaIaem4Ba8MaemOzayMaeeiiaaIaemiDaqNaemyAaKMaemyBa0MaemyzauMaeeiiaaIae8xAaKMae8NBa4Mae8hDaqNaemyzauMaemOCaiNaemODayNaemyyaeMaemiBaWMaeyOeI0Iaem4uamLaemiDaqNaemyyaeMaemOCaiNaemiDaqNaeeiiaaIaem4Ba8MaemOzayMaeeiiaaIaemiDaqNaemyAaKMaemyBa0MaemyzauMaeeiiaaIae8xAaKMae8NBa4Mae8hDaqNaemyzauMaemOCaiNaemODayNaemyyaeMaemiBaWMaeiykaKcabaGaeiikaGIaemyta0KaemyyaeMaemiEaGNaemyAaKMaemyBa0MaemyDauNaemyBa0MaeeiiaaIaemOzayMaem4Ba8MaemiBaWMaemiBaWMaem4Ba8Maem4DaCNaeeiiaaIaemyDauNaemiCaaNaeyOeI0Iaem4uamLaemiDaqNaemyyaeMaemOCaiNaemiDaqNaeeiiaaIaem4Ba8MaemOzayMaeeiiaaIaemiDaqNaemyAaKMaemyBa0MaemyzauMaeeiiaaIae8xAaKMae8NBa4Mae8hDaqNaemyzauMaemOCaiNaemODayNaemyyaeMaemiBaWMaeiykaKcaaaGaayjkaiaawMcaaaaa@CA56@

Using the data from step 1, the estimated maximum follow-up of 82 months and equation (18):

Censored (15−18 month), control=339.50×(12×(18−15)(82−15))=7.60Censored (15−18 month), research=358.43×(12×(18−15)(82−15))=8.02
 MathType@MTEF@5@5@+=feaafiart1ev1aaatCvAUfKttLearuWrP9MDH5MBPbIqV92AaeXatLxBI9gBaebbnrfifHhDYfgasaacH8akY=wiFfYdH8Gipec8Eeeu0xXdbba9frFj0=OqFfea0dXdd9vqai=hGuQ8kuc9pgc9s8qqaq=dirpe0xb9q8qiLsFr0=vr0=vr0dc8meaabaqaciaacaGaaeqabaqabeGadaaakeaafaqaaeGabaaabaGaem4qamKaemyzauMaemOBa4Maem4CamNaem4Ba8MaemOCaiNaemyzauMaemizaqMaeeiiaaIaeiikaGIaeGymaeJaeGynauJaeyOeI0IaeGymaeJaeGioaGJaeeiiaaIaemyBa0Maem4Ba8MaemOBa4MaemiDaqNaemiAaGMaeiykaKIaeiilaWIaeeiiaaIaem4yamMaem4Ba8MaemOBa4MaemiDaqNaemOCaiNaem4Ba8MaemiBaWMaeyypa0JaeG4mamJaeG4mamJaeGyoaKJaeiOla4IaeGynauJaeGimaaJaey41aq7aaeWaaeaadaWcaaqaaiabigdaXaqaaiabikdaYaaacqGHxdaTdaWcaaqaaiabcIcaOiabigdaXiabiIda4iabgkHiTiabigdaXiabiwda1iabcMcaPaqaaiabcIcaOiabiIda4iabikdaYiabgkHiTiabigdaXiabiwda1iabcMcaPaaaaiaawIcacaGLPaaacqGH9aqpcqaI3aWncqGGUaGlcqaI2aGncqaIWaamaeaacqWGdbWqcqWGLbqzcqWGUbGBcqWGZbWCcqWGVbWBcqWGYbGCcqWGLbqzcqWGKbazcqqGGaaicqGGOaakcqaIXaqmcqaI1aqncqGHsislcqaIXaqmcqaI4aaocqqGGaaicqWGTbqBcqWGVbWBcqWGUbGBcqWG0baDcqWGObaAcqGGPaqkcqGGSaalcqqGGaaicqWGYbGCcqWGLbqzcqWGZbWCcqWGLbqzcqWGHbqycqWGYbGCcqWGJbWycqWGObaAcqGH9aqpcqaIZaWmcqaI1aqncqaI4aaocqGGUaGlcqaI0aancqaIZaWmcqGHxdaTdaqadaqaamaalaaabaGaeGymaedabaGaeGOmaidaaiabgEna0oaalaaabaGaeiikaGIaeGymaeJaeGioaGJaeyOeI0IaeGymaeJaeGynauJaeiykaKcabaGaeiikaGIaeGioaGJaeGOmaiJaeyOeI0IaeGymaeJaeGynauJaeiykaKcaaaGaayjkaiaawMcaaiabg2da9iabiIda4iabc6caUiabicdaWiabikdaYaaaaaa@B853@

around 8 patients in the research arm and 7 patients in the control arm were estimated to be censored during 15–18 month time interval:

#### Step 3. Numbers at risk during the current interval, adjusted for censoring

The numbers censored can be used to adjust (reduce) the numbers at risk during the time interval:

*At risk during current interval, adjusted for censoring *= *Event free at start of current interval *- *Censored during current interval*

Based on the data from step 1 and 2, the numbers at risk during the current 15–18 month time interval are:

*At risk during, adjusted for censoring (15 – 18 month), research *= 358.43 - 8.02 = 350.41

*At risk during, adjusted for censoring (15 – 18 month), control = 339.50 -7.60 = 331.90*

#### Step 4. Number of events during the current interval

The number of events during the interval is then estimated from the reduced numbers at risk:

Events in current interval=At risk during current interval×(% Event free at start−% Event free at end% Event free at start)
 MathType@MTEF@5@5@+=feaafiart1ev1aaatCvAUfKttLearuWrP9MDH5MBPbIqV92AaeXatLxBI9gBaebbnrfifHhDYfgasaacH8akY=wiFfYdH8Gipec8Eeeu0xXdbba9frFj0=OqFfea0dXdd9vqai=hGuQ8kuc9pgc9s8qqaq=dirpe0xb9q8qiLsFr0=vr0=vr0dc8meaabaqaciaacaGaaeqabaqabeGadaaakeaacqWGfbqrcqWG2bGDcqWGLbqzcqWGUbGBcqWG0baDieGacqWFZbWCcqqGGaaicqWFPbqAcqWFUbGBcqqGGaaicqWGJbWycqWG1bqDcqWGYbGCcqWGYbGCcqWGLbqzcqWGUbGBcqWG0baDcqqGGaaicqWFPbqAcqWFUbGBcqWF0baDcqWGLbqzcqWGYbGCcqWG2bGDcqWGHbqycqWGSbaBcqGH9aqpcqWGbbqqcqWG0baDcqqGGaaicqWGYbGCcqWGPbqAcqWGZbWCcqWGRbWAcqqGGaaicqWGKbazcqWG1bqDcqWGYbGCcqWGPbqAcqWGUbGBcqWGNbWzcqqGGaaicqWGJbWycqWG1bqDcqWGYbGCcqWGYbGCcqWGLbqzcqWGUbGBcqWG0baDcqqGGaaicqWFPbqAcqWFUbGBcqWF0baDcqWGLbqzcqWGYbGCcqWG2bGDcqWGHbqycqWGSbaBcqGHxdaTdaqadaqaamaalaaabaGaeiyjauIaeeiiaaIaemyrauKaemODayNaemyzauMaemOBa4MaemiDaqNaeeiiaaIaemOzayMaemOCaiNaemyzauMaemyzauMaeeiiaaIaemyyaeMaemiDaqNaeeiiaaIaem4CamNaemiDaqNaemyyaeMaemOCaiNaemiDaqNaeyOeI0IaeiyjauIaeeiiaaIaemyrauKaemODayNaemyzauMaemOBa4MaemiDaqNaeeiiaaIaemOzayMaemOCaiNaemyzauMaemyzauMaeeiiaaIaemyyaeMaemiDaqNaeeiiaaIaemyzauMaemOBa4MaemizaqgabaGaeiyjauIaeeiiaaIaemyrauKaemODayNaemyzauMaemOBa4MaemiDaqNaeeiiaaIaemOzayMaemOCaiNaemyzauMaemyzauMaeeiiaaIaemyyaeMaemiDaqNaeeiiaaIaem4CamNaemiDaqNaemyyaeMaemOCaiNaemiDaqhaaaGaayjkaiaawMcaaaaa@C6F3@

Using the numbers at risk during the interval from step 3 and the data extracted from the curve (Table 2) in equation (20), allows estimation of the number of events in the 15–18 month interval:

Events during (15−18 month), research=350.41×(73−6873)=24.00Events during (15−18 month), control=331.90×(70−6370)=33.19
 MathType@MTEF@5@5@+=feaafiart1ev1aaatCvAUfKttLearuWrP9MDH5MBPbIqV92AaeXatLxBI9gBaebbnrfifHhDYfgasaacH8akY=wiFfYdH8Gipec8Eeeu0xXdbba9frFj0=OqFfea0dXdd9vqai=hGuQ8kuc9pgc9s8qqaq=dirpe0xb9q8qiLsFr0=vr0=vr0dc8meaabaqaciaacaGaaeqabaqabeGadaaakeaafaqaaeGabaaabaGaemyrauKaemODayNaemyzauMaemOBa4MaemiDaqNaem4CamNaeeiiaaIaemizaqMaemyDauNaemOCaiNaemyAaKMaemOBa4Maem4zaCMaeeiiaaIaeiikaGIaeGymaeJaeGynauJaeyOeI0IaeGymaeJaeGioaGJaeeiiaaIaemyBa0Maem4Ba8MaemOBa4MaemiDaqNaemiAaGMaeiykaKIaeiilaWIaeeiiaaIaemOCaiNaemyzauMaem4CamNaemyzauMaemyyaeMaemOCaiNaem4yamMaemiAaGMaeyypa0JaeG4mamJaeGynauJaeGimaaJaeiOla4IaeGinaqJaeGymaeJaey41aq7aaeWaaeaadaWcaaqaaiabiEda3iabiodaZiabgkHiTiabiAda2iabiIda4aqaaiabiEda3iabiodaZaaaaiaawIcacaGLPaaacqGH9aqpcqaIYaGmcqaI0aancqGGUaGlcqaIWaamcqaIWaamaeaacqWGfbqrcqWG2bGDcqWGLbqzcqWGUbGBcqWG0baDcqWGZbWCcqqGGaaicqWGKbazcqWG1bqDcqWGYbGCcqWGPbqAcqWGUbGBcqWGNbWzcqqGGaaicqGGOaakcqaIXaqmcqaI1aqncqGHsislcqaIXaqmcqaI4aaocqqGGaaicqWGTbqBcqWGVbWBcqWGUbGBcqWG0baDcqWGObaAcqGGPaqkcqGGSaalcqqGGaaicqWGJbWycqWGVbWBcqWGUbGBcqWG0baDcqWGYbGCcqWGVbWBcqWGSbaBcqGH9aqpcqaIZaWmcqaIZaWmcqaIXaqmcqGGUaGlcqaI5aqocqaIWaamcqGHxdaTdaqadaqaamaalaaabaGaeG4naCJaeGimaaJaeyOeI0IaeGOnayJaeG4mamdabaGaeG4naCJaeGimaadaaaGaayjkaiaawMcaaiabg2da9iabiodaZiabiodaZiabc6caUiabigdaXiabiMda5aaaaaa@B29D@

#### Step 5. Estimate the HR, V and O-E for the current interval

As time to event and censoring have already been accounted for, the hazard ratio can be estimated by using the equation for calculating a relative risk:

HR=(Events research/At risk researchEvents control/At risk control)
 MathType@MTEF@5@5@+=feaafiart1ev1aaatCvAUfKttLearuWrP9MDH5MBPbIqV92AaeXatLxBI9gBaebbnrfifHhDYfgasaacH8akY=wiFfYdH8Gipec8Eeeu0xXdbba9frFj0=OqFfea0dXdd9vqai=hGuQ8kuc9pgc9s8qqaq=dirpe0xb9q8qiLsFr0=vr0=vr0dc8meaabaqaciaacaGaaeqabaqabeGadaaakeaacqqGibascqqGsbGucqGH9aqpdaqadaqaamaalaaabaGaemyrauKaemODayNaemyzauMaemOBa4MaemiDaqNaem4CamNaeeiiaaIaemOCaiNaemyzauMaem4CamNaemyzauMaemyyaeMaemOCaiNaem4yamMaemiAaGMaei4la8IaemyqaeKaemiDaqNaeeiiaaIaemOCaiNaemyAaKMaem4CamNaem4AaSMaeeiiaaIaemOCaiNaemyzauMaem4CamNaemyzauMaemyyaeMaemOCaiNaem4yamMaemiAaGgabaGaemyrauKaemODayNaemyzauMaemOBa4MaemiDaqNaem4CamNaeeiiaaIaem4yamMaem4Ba8MaemOBa4MaemiDaqNaemOCaiNaem4Ba8MaemiBaWMaei4la8IaemyqaeKaemiDaqNaeeiiaaIaemOCaiNaemyAaKMaem4CamNaem4AaSMaeeiiaaIaem4yamMaem4Ba8MaemOBa4MaemiDaqNaemOCaiNaem4Ba8MaemiBaWgaaaGaayjkaiaawMcaaaaa@81AD@

with associated *V*:

V=1[(1/Events research)−(1/At risk research)+(1/Events control)−(1/Events control)]
 MathType@MTEF@5@5@+=feaafiart1ev1aaatCvAUfKttLearuWrP9MDH5MBPbIqV92AaeXatLxBI9gBaebbnrfifHhDYfgasaacH8akY=wiFfYdH8Gipec8Eeeu0xXdbba9frFj0=OqFfea0dXdd9vqai=hGuQ8kuc9pgc9s8qqaq=dirpe0xb9q8qiLsFr0=vr0=vr0dc8meaabaqaciaacaGaaeqabaqabeGadaaakeaacqWGwbGvcqGH9aqpdaWcaaqaaiabigdaXaqaaiabcUfaBjabcIcaOiabigdaXiabc+caViabdweafjabdAha2jabdwgaLjabd6gaUjabdsha0jabdohaZjabbccaGiabdkhaYjabdwgaLjabdohaZjabdwgaLjabdggaHjabdkhaYjabdogaJjabdIgaOjabcMcaPiabgkHiTiabcIcaOiabigdaXiabc+caViabdgeabjabdsha0jabbccaGiabdkhaYjabdMgaPjabdohaZjabdUgaRjabbccaGiabdkhaYjabdwgaLjabdohaZjabdwgaLjabdggaHjabdkhaYjabdogaJjabdIgaOjabcMcaPiabgUcaRiabcIcaOiabigdaXiabc+caViabdweafjabdAha2jabdwgaLjabd6gaUjabdsha0jabdohaZjabbccaGiabdogaJjabd+gaVjabd6gaUjabdsha0jabdkhaYjabd+gaVjabdYgaSjabcMcaPiabgkHiTiabcIcaOiabigdaXiabc+caViabdweafjabdAha2jabdwgaLjabd6gaUjabdsha0jabdohaZjabbccaGiabdogaJjabd+gaVjabd6gaUjabdsha0jabdkhaYjabd+gaVjabdYgaSjabcMcaPiabc2faDbaaaaa@90DE@

Using the data from steps 3 and 5 and equations (21), (22) and (8) above, but without rounding:

HR=[24.00/350.4133.19/331.90]=0.68V=1[1/24.0−1/350.41+1/33.19−1/331.90]=15.17
 MathType@MTEF@5@5@+=feaafiart1ev1aaatCvAUfKttLearuWrP9MDH5MBPbIqV92AaeXatLxBI9gBaebbnrfifHhDYfgasaacH8akY=wiFfYdH8Gipec8Eeeu0xXdbba9frFj0=OqFfea0dXdd9vqai=hGuQ8kuc9pgc9s8qqaq=dirpe0xb9q8qiLsFr0=vr0=vr0dc8meaabaqaciaacaGaaeqabaqabeGadaaakeaafaqabeGabaaabaGaeeisaGKaeeOuaiLaeyypa0ZaamWaaeaadaWcaaqaaiabikdaYiabisda0iabc6caUiabicdaWiabicdaWiabc+caViabiodaZiabiwda1iabicdaWiabc6caUiabisda0iabigdaXaqaaiabiodaZiabiodaZiabc6caUiabigdaXiabiMda5iabc+caViabiodaZiabiodaZiabigdaXiabc6caUiabiMda5iabicdaWaaaaiaawUfacaGLDbaacqGH9aqpcqaIWaamcqGGUaGlcqaI2aGncqaI4aaoaeaacqWGwbGvcqGH9aqpdaWcaaqaaiabigdaXaqaaiabcUfaBjabigdaXiabc+caViabikdaYiabisda0iabc6caUiabicdaWiabgkHiTiabigdaXiabc+caViabiodaZiabiwda1iabicdaWiabc6caUiabisda0iabigdaXiabgUcaRiabigdaXiabc+caViabiodaZiabiodaZiabc6caUiabigdaXiabiMda5iabgkHiTiabigdaXiabc+caViabiodaZiabiodaZiabigdaXiabc6caUiabiMda5iabicdaWiabc2faDbaacqGH9aqpcqaIXaqmcqaI1aqncqGGUaGlcqaIXaqmcqaI3aWnaaaaaa@76A2@

*O - E *= ln(0.68) × 15.17 = -5.74

Gives estimates of the HR, *V* and *O-E* as 0.68, 15.17 and -5.74, respectively for the 15–18 month time interval. Note that if censoring had not been taken into account, the estimate of the HR for this time interval would still have been 0.68, but the *V *would be slightly greater at 15.52.

These steps are repeated for all time intervals.

#### Step 6, combining all time intervals

The final step is to calculate the overall HR for the trial using the formula for calculating a pooled HR shown previously (1). Taking all time intervals and accounting for censoring a pooled HR of 0.88 and *V *of 128.81 (95%CI of 0.74–1.05) is obtained:

HR=exp⁡[∑O−E∑V]=exp⁡[[(0.00)+(0.00)+(−5.21)+(−3.25)+(−0.51)+(−5.74)...etc.][(7.55)+(12.86)+(18.10)+(22.96)+(13.05)+(15.17)...etc.]]=exp⁡[−16.35128.81]HR=0.88
 MathType@MTEF@5@5@+=feaafiart1ev1aaatCvAUfKttLearuWrP9MDH5MBPbIqV92AaeXatLxBI9gBaebbnrfifHhDYfgasaacH8akY=wiFfYdH8Gipec8Eeeu0xXdbba9frFj0=OqFfea0dXdd9vqai=hGuQ8kuc9pgc9s8qqaq=dirpe0xb9q8qiLsFr0=vr0=vr0dc8meaabaqaciaacaGaaeqabaqabeGadaaakeaafaqadeabbaaaaeaacqqGibascqqGsbGucqGH9aqpcyGGLbqzcqGG4baEcqGGWbaCdaWadaqaamaalaaabaWaaabqaeaacqWGpbWtcqGHsislcqWGfbqraSqabeqaniabggHiLdaakeaadaaeabqaaiabdAfawbWcbeqab0GaeyyeIuoaaaaakiaawUfacaGLDbaaaeaacqGH9aqpcyGGLbqzcqGG4baEcqGGWbaCdaWadaqaamaalaaabaGaei4waSLaeiikaGIaeGimaaJaeiOla4IaeGimaaJaeGimaaJaeiykaKIaey4kaSIaeiikaGIaeGimaaJaeiOla4IaeGimaaJaeGimaaJaeiykaKIaey4kaSIaeiikaGIaeyOeI0IaeGynauJaeiOla4IaeGOmaiJaeGymaeJaeiykaKIaey4kaSIaeiikaGIaeyOeI0IaeG4mamJaeiOla4IaeGOmaiJaeGynauJaeiykaKIaey4kaSIaeiikaGIaeyOeI0IaeGimaaJaeiOla4IaeGynauJaeGymaeJaeiykaKIaey4kaSIaeiikaGIaeyOeI0IaeGynauJaeiOla4IaeG4naCJaeGinaqJaeiykaKIaeiOla4IaeiOla4IaeiOla4IaemyzauMaemiDaqNaem4yamMaeiOla4Iaeiyxa0fabaGaei4waSLaeiikaGIaeG4naCJaeiOla4IaeGynauJaeGynauJaeiykaKIaey4kaSIaeiikaGIaeGymaeJaeGOmaiJaeiOla4IaeGioaGJaeGOnayJaeiykaKIaey4kaSIaeiikaGIaeGymaeJaeGioaGJaeiOla4IaeGymaeJaeGimaaJaeiykaKIaey4kaSIaeiikaGIaeGOmaiJaeGOmaiJaeiOla4IaeGyoaKJaeGOnayJaeiykaKIaey4kaSIaeiikaGIaeGymaeJaeG4mamJaeiOla4IaeGimaaJaeGynauJaeiykaKIaey4kaSIaeiikaGIaeGymaeJaeGynauJaeiOla4IaeGymaeJaeG4naCJaeiykaKIaeiOla4IaeiOla4IaeiOla4IaemyzauMaemiDaqNaem4yamMaeiOla4Iaeiyxa0faaaGaay5waiaaw2faaaqaaiabg2da9iGbcwgaLjabcIha4jabcchaWnaadmaabaWaaSaaaeaacqGHsislcqaIXaqmcqaI2aGncqGGUaGlcqaIZaWmcqaI1aqnaeaacqaIXaqmcqaIYaGmcqaI4aaocqGGUaGlcqaI4aaocqaIXaqmaaaacaGLBbGaayzxaaaabaGaeeisaGKaeeOuaiLaeyypa0JaeGimaaJaeiOla4IaeGioaGJaeGioaGdaaaaa@C6AC@

In this example, if the censoring model had not been applied the same HR, a smaller, but similar *V *(136.23) and a similar CI (0.74–1.04) would have been estimated. This is probably because it is a large trial with good follow-up, making both estimates fairly precise. In contrast, the ovarian cancer trial [5] accrued far fewer patients and had poorer follow-up. Using the curve method and accounting for censoring, gives a HR estimate of 1.21 (95% CI 0.62–2.36), but discounting censoring, the HR is slightly more extreme (1.26), with overly precise confidence intervals (95% CI 0.69–2.28). I n other situations the differences may be more pronounced.

### 11. Report presents Kaplan-Meier curve and the numbers at risk

The presentation of the numbers at risk at particular time points with a Kaplan-Meier curve, offers a more direct means of assessing the level of censoring [2], which is taken into account when the HR, *V *and *O-E *are estimated. However, this necessarily limits the division of the curve to these time points, which may be relatively few. Further this approach may be problematic when the event rate between time points is large, e.g. greater than 20% [1].

The number of patients event-free at each time point i.e. the numbers of patients event-free at the start and end of the each time interval is known, and so they do not need to be estimated. For each time interval for each arm, assuming that the level of censoring is constant within each interval, it remains to calculate the number of patients who were: 1) at risk during the interval and 2) the number of events during the interval. These can be used to 4) estimate the *O-E*, *V *and HR for the time interval and the data from all the intervals can be combined in 5) to obtain the *O-E*, *V *and HR for the complete curve. Although not required to estimate the HR, the number of patients who were 3) censored during the interval can also be calculated and is useful for comparison with the other curve method.

The bladder cancer trial report gave the numbers at risk annually until 5 years. These data, and the percentage survival (i.e. event-free) for each arm at the start of each time interval, are given in Table 2 and can be used to illustrate the steps of the method for the 0–12 month time period:

#### Step 1. Numbers at risk during the current interval

The same data can be used to quantify the numbers of patients at risk during an interval:

At risk during current interval=(At risk at start+At risk at end)×% Event free at start(% Event free at start+% Event free at end)
 MathType@MTEF@5@5@+=feaafiart1ev1aaatCvAUfKttLearuWrP9MDH5MBPbIqV92AaeXatLxBI9gBaebbnrfifHhDYfgasaacH8akY=wiFfYdH8Gipec8Eeeu0xXdbba9frFj0=OqFfea0dXdd9vqai=hGuQ8kuc9pgc9s8qqaq=dirpe0xb9q8qiLsFr0=vr0=vr0dc8meaabaqaciaacaGaaeqabaqabeGadaaakeaacqWGbbqqcqWG0baDcqqGGaaicqWGYbGCcqWGPbqAcqWGZbWCcqWGRbWAcqqGGaaicqWGKbazcqWG1bqDcqWGYbGCcqWGPbqAcqWGUbGBcqWGNbWzcqqGGaaicqWGJbWycqWG1bqDcqWGYbGCcqWGYbGCcqWGLbqzcqWGUbGBcqWG0baDcqqGGaaiieGacqWFPbqAcqWFUbGBcqWF0baDcqWGLbqzcqWGYbGCcqWG2bGDcqWGHbqycqWGSbaBcqGH9aqpdaWcaaqaaiabcIcaOiabdgeabjabdsha0jabbccaGiabdkhaYjabdMgaPjabdohaZjabdUgaRjabbccaGiabdggaHjabdsha0jabbccaGiabdohaZjabdsha0jabdggaHjabdkhaYjabdsha0jabgUcaRiabdgeabjabdsha0jabbccaGiabdkhaYjabdMgaPjabdohaZjabdUgaRjabbccaGiabdggaHjabdsha0jabbccaGiabdwgaLjabd6gaUjabdsgaKjabcMcaPiabgEna0kabcwcaLiabbccaGiabdweafjabdAha2jabdwgaLjabd6gaUjabdsha0jabbccaGiabdAgaMjabdkhaYjabdwgaLjabdwgaLjabbccaGiabdggaHjabdsha0jabbccaGiabdohaZjabdsha0jabdggaHjabdkhaYjabdsha0bqaaiabcIcaOiabcwcaLiabbccaGiabdweafjabdAha2jabdwgaLjabd6gaUjabdsha0jabbccaGiabdAgaMjabdkhaYjabdwgaLjabdwgaLjabbccaGiabdggaHjabdsha0jabbccaGiabdohaZjabdsha0jabdggaHjabdkhaYjabdsha0jabgUcaRiabcwcaLiabbccaGiabdweafjabdAha2jabdwgaLjabd6gaUjabdsha0jabbccaGiabdAgaMjabdkhaYjabdwgaLjabdwgaLjabbccaGiabdggaHjabdsha0jabbccaGiabdwgaLjabd6gaUjabdsgaKjabcMcaPaaaaaa@CD09@

For the 0–12 month interval:

At risk during (0−12 month), research=(491+372)×100(100+78)=484.83At risk during (0−12 month), control=(485+355)×100(100+75)=480.00
 MathType@MTEF@5@5@+=feaafiart1ev1aaatCvAUfKttLearuWrP9MDH5MBPbIqV92AaeXatLxBI9gBaebbnrfifHhDYfgasaacH8akY=wiFfYdH8Gipec8Eeeu0xXdbba9frFj0=OqFfea0dXdd9vqai=hGuQ8kuc9pgc9s8qqaq=dirpe0xb9q8qiLsFr0=vr0=vr0dc8meaabaqaciaacaGaaeqabaqabeGadaaakeaafaqaaeGabaaabaGaemyqaeKaemiDaqNaeeiiaaIaemOCaiNaemyAaKMaem4CamNaem4AaSMaeeiiaaIaemizaqMaemyDauNaemOCaiNaemyAaKMaemOBa4Maem4zaCMaeeiiaaIaeiikaGIaeGimaaJaeyOeI0IaeGymaeJaeGOmaiJaeeiiaaIaemyBa0Maem4Ba8MaemOBa4MaemiDaqNaemiAaGMaeiykaKIaeiilaWIaeeiiaaIaemOCaiNaemyzauMaem4CamNaemyzauMaemyyaeMaemOCaiNaem4yamMaemiAaGMaeyypa0ZaaSaaaeaacqGGOaakcqaI0aancqaI5aqocqaIXaqmcqGHRaWkcqaIZaWmcqaI3aWncqaIYaGmcqGGPaqkcqGHxdaTcqaIXaqmcqaIWaamcqaIWaamaeaacqGGOaakcqaIXaqmcqaIWaamcqaIWaamcqGHRaWkcqaI3aWncqaI4aaocqGGPaqkaaGaeyypa0JaeGinaqJaeGioaGJaeGinaqJaeiOla4IaeGioaGJaeG4mamdabaGaemyqaeKaemiDaqNaeeiiaaIaemOCaiNaemyAaKMaem4CamNaem4AaSMaeeiiaaIaemizaqMaemyDauNaemOCaiNaemyAaKMaemOBa4Maem4zaCMaeeiiaaIaeiikaGIaeGimaaJaeyOeI0IaeGymaeJaeGOmaiJaeeiiaaIaemyBa0Maem4Ba8MaemOBa4MaemiDaqNaemiAaGMaeiykaKIaeiilaWIaeeiiaaIaem4yamMaem4Ba8MaemOBa4MaemiDaqNaemOCaiNaem4Ba8MaemiBaWMaeyypa0ZaaSaaaeaacqGGOaakcqaI0aancqaI4aaocqaI1aqncqGHRaWkcqaIZaWmcqaI1aqncqaI1aqncqGGPaqkcqGHxdaTcqaIXaqmcqaIWaamcqaIWaamaeaacqGGOaakcqaIXaqmcqaIWaamcqaIWaamcqGHRaWkcqaI3aWncqaI1aqncqGGPaqkaaGaeyypa0JaeGinaqJaeGioaGJaeGimaaJaeiOla4IaeGimaaJaeGimaadaaaaa@BD33@

#### Step 2. Number of events during the current interval

Again, the same published data can be used to estimate the number of events in an interval:

Events in current interval=(At risk at start+At risk at end)(% Event free at start−% Event free at end)(% Event free at start+% Event free at end)
 MathType@MTEF@5@5@+=feaafiart1ev1aaatCvAUfKttLearuWrP9MDH5MBPbIqV92AaeXatLxBI9gBamXvP5wqSXMqHnxAJn0BKvguHDwzZbqegyvzYrwyUfgarqqtubsr4rNCHbGeaGqiA8vkIkVAFgIELiFeLkFeLk=iY=Hhbbf9v8qqaqFr0xc9pk0xbba9q8WqFfeaY=biLkVcLq=JHqVepeea0=as0db9vqpepesP0xe9Fve9Fve9GapdbaqaaeGacaGaaiaabeqaamqadiabaaGcbaGaemyrauKaemODayNaemyzauMaemOBa4MaemiDaqhcdiGaa83CaiabbccaGiaa=LgacaWFUbGaeeiiaaIaem4yamMaemyDauNaemOCaiNaemOCaiNaemyzauMaemOBa4MaemiDaqNaeeiiaaIaa8xAaiaa=5gacaWF0bGaemyzauMaemOCaiNaemODayNaemyyaeMaemiBaWMaeyypa0ZaaSaaaeaacqGGOaakcqWGbbqqcqWG0baDcqqGGaaicqWGYbGCcqWGPbqAcqWGZbWCcqWGRbWAcqqGGaaicqWGHbqycqWG0baDcqqGGaaicqWGZbWCcqWG0baDcqWGHbqycqWGYbGCcqWG0baDcqGHRaWkcqWGbbqqcqWG0baDcqqGGaaicqWGYbGCcqWGPbqAcqWGZbWCcqWGRbWAcqqGGaaicqWGHbqycqWG0baDcqqGGaaicqWGLbqzcqWGUbGBcqWGKbazcqGGPaqkcqGGOaakcqGGLaqjcqqGGaaicqWGfbqrcqWG2bGDcqWGLbqzcqWGUbGBcqWG0baDcqqGGaaicqWGMbGzcqWGYbGCcqWGLbqzcqWGLbqzcqqGGaaicqWGHbqycqWG0baDcqqGGaaicqWGZbWCcqWG0baDcqWGHbqycqWGYbGCcqWG0baDcqGHsislcqGGLaqjcqqGGaaicqWGfbqrcqWG2bGDcqWGLbqzcqWGUbGBcqWG0baDcqqGGaaicqWGMbGzcqWGYbGCcqWGLbqzcqWGLbqzcqqGGaaicqWGHbqycqWG0baDcqqGGaaicqWGLbqzcqWGUbGBcqWGKbazcqGGPaqkaeaacqGGOaakcqGGLaqjcqqGGaaicqWGfbqrcqWG2bGDcqWGLbqzcqWGUbGBcqWG0baDcqqGGaaicqWGMbGzcqWGYbGCcqWGLbqzcqWGLbqzcqqGGaaicqWGHbqycqWG0baDcqqGGaaicqWGZbWCcqWG0baDcqWGHbqycqWGYbGCcqWG0baDcqGHRaWkcqGGLaqjcqqGGaaicqWGfbqrcqWG2bGDcqWGLbqzcqWGUbGBcqWG0baDcqqGGaaicqWGMbGzcqWGYbGCcqWGLbqzcqWGLbqzcqqGGaaicqWGHbqycqWG0baDcqqGGaaicqWGLbqzcqWGUbGBcqWGKbazcqGGPaqkaaaaaa@EBA4@

For the 0–12 month interval:

Events during (0−12 month), research=(491+372)(100−78)(100+78)=106.67Events during (0−12 month), research=(485+355)(100−75)(100+75)=120.00
 MathType@MTEF@5@5@+=feaafiart1ev1aaatCvAUfKttLearuWrP9MDH5MBPbIqV92AaeXatLxBI9gBaebbnrfifHhDYfgasaacH8akY=wiFfYdH8Gipec8Eeeu0xXdbba9frFj0=OqFfea0dXdd9vqai=hGuQ8kuc9pgc9s8qqaq=dirpe0xb9q8qiLsFr0=vr0=vr0dc8meaabaqaciaacaGaaeqabaqabeGadaaakeaafaqaaeGabaaabaGaemyrauKaemODayNaemyzauMaemOBa4MaemiDaqNaem4CamNaeeiiaaIaemizaqMaemyDauNaemOCaiNaemyAaKMaemOBa4Maem4zaCMaeeiiaaIaeiikaGIaeGimaaJaeyOeI0IaeGymaeJaeGOmaiJaeeiiaaIaemyBa0Maem4Ba8MaemOBa4MaemiDaqNaemiAaGMaeiykaKIaeiilaWIaeeiiaaIaemOCaiNaemyzauMaem4CamNaemyzauMaemyyaeMaemOCaiNaem4yamMaemiAaGMaeyypa0ZaaSaaaeaacqGGOaakcqaI0aancqaI5aqocqaIXaqmcqGHRaWkcqaIZaWmcqaI3aWncqaIYaGmcqGGPaqkcqGGOaakcqaIXaqmcqaIWaamcqaIWaamcqGHsislcqaI3aWncqaI4aaocqGGPaqkaeaacqGGOaakcqaIXaqmcqaIWaamcqaIWaamcqGHRaWkcqaI3aWncqaI4aaocqGGPaqkaaGaeyypa0JaeGymaeJaeGimaaJaeGOnayJaeiOla4IaeGOnayJaeG4naCdabaGaemyrauKaemODayNaemyzauMaemOBa4MaemiDaqNaem4CamNaeeiiaaIaemizaqMaemyDauNaemOCaiNaemyAaKMaemOBa4Maem4zaCMaeeiiaaIaeiikaGIaeGimaaJaeyOeI0IaeGymaeJaeGOmaiJaeeiiaaIaemyBa0Maem4Ba8MaemOBa4MaemiDaqNaemiAaGMaeiykaKIaeiilaWIaeeiiaaIaemOCaiNaemyzauMaem4CamNaemyzauMaemyyaeMaemOCaiNaem4yamMaemiAaGMaeyypa0ZaaSaaaeaacqGGOaakcqaI0aancqaI4aaocqaI1aqncqGHRaWkcqaIZaWmcqaI1aqncqaI1aqncqGGPaqkcqGGOaakcqaIXaqmcqaIWaamcqaIWaamcqGHsislcqaI3aWncqaI1aqncqGGPaqkaeaacqGGOaakcqaIXaqmcqaIWaamcqaIWaamcqGHRaWkcqaI3aWncqaI1aqncqGGPaqkaaGaeyypa0JaeGymaeJaeGOmaiJaeGimaaJaeiOla4IaeGimaaJaeGimaadaaaaa@C1C0@

There were approximately 106 events estimated on the research arm and 120 on the control arm.

#### Step 3. Numbers censored during the current interval

The numbers censored are obtained from the reported numbers at risk and the event rate at the start and end of an interval:

Censored during currentinterval=2×(At risk at start×% Event free at end−At risk at end×% Event free at start)(% Event free at start+% Event free at end)
 MathType@MTEF@5@5@+=feaafiart1ev1aaatCvAUfKttLearuWrP9MDH5MBPbIqV92AaeXatLxBI9gBaebbnrfifHhDYfgasaacH8akY=wiFfYdH8Gipec8Eeeu0xXdbba9frFj0=OqFfea0dXdd9vqai=hGuQ8kuc9pgc9s8qqaq=dirpe0xb9q8qiLsFr0=vr0=vr0dc8meaabaqaciaacaGaaeqabaqabeGadaaakeaacqWGdbWqcqWGLbqzcqWGUbGBcqWGZbWCcqWGVbWBcqWGYbGCcqWGLbqzcqWGKbazcqqGGaaicqWGKbazcqWG1bqDcqWGYbGCcqWGPbqAcqWGUbGBcqWGNbWzcqqGGaaicqWGJbWycqWG1bqDcqWGYbGCcqWGYbGCcqWGLbqzcqWGUbGBcqWG0baDieaacqWFGaaiieGacqGFPbqAcqGFUbGBcqGF0baDcqWGLbqzcqWGYbGCcqWG2bGDcqWGHbqycqWGSbaBcqGH9aqpdaWcaaqaaiabikdaYiabgEna0kabcIcaOiabdgeabjabdsha0jabbccaGiabdkhaYjabdMgaPjabdohaZjabdUgaRjabbccaGiabdggaHjabdsha0jabbccaGiabdohaZjabdsha0jabdggaHjabdkhaYjabdsha0jabgEna0kabcwcaLiabbccaGiabdweafjabdAha2jabdwgaLjabd6gaUjabdsha0jabbccaGiabdAgaMjabdkhaYjabdwgaLjabdwgaLjabbccaGiabdggaHjabdsha0jabbccaGiabdwgaLjabd6gaUjabdsgaKjabgkHiTiabdgeabjabdsha0jabbccaGiabdkhaYjabdMgaPjabdohaZjabdUgaRjabbccaGiabdggaHjabdsha0jabbccaGiabdwgaLjabd6gaUjabdsgaKjabgEna0kabcwcaLiabbccaGiabdweafjabdAha2jabdwgaLjabd6gaUjabdsha0jabbccaGiabdAgaMjabdkhaYjabdwgaLjabdwgaLjabbccaGiabdggaHjabdsha0jabbccaGiabdohaZjabdsha0jabdggaHjabdkhaYjabdsha0jabcMcaPaqaaiabcIcaOiabcwcaLiabbccaGiabdweafjabdAha2jabdwgaLjabd6gaUjabdsha0jabbccaGiabdAgaMjabdkhaYjabdwgaLjabdwgaLjabbccaGiabdggaHjabdsha0jabbccaGiabdohaZjabdsha0jabdggaHjabdkhaYjabdsha0jabgUcaRiabcwcaLiabbccaGiabdweafjabdAha2jabdwgaLjabd6gaUjabdsha0jabbccaGiabdAgaMjabdkhaYjabdwgaLjabdwgaLjabbccaGiabdggaHjabdsha0jabbccaGiabdwgaLjabd6gaUjabdsgaKjabcMcaPaaaaaa@EAD9@

Using event rates extracted from the curve at 0 and 12 months and the associated numbers at risk:

Censored during (0−12 month), research=2×(491×78−372×100)(100+78)=12.33Censored during (0−12 month), control=2×(485×75−385×100)(100+75)=10.00
 MathType@MTEF@5@5@+=feaafiart1ev1aaatCvAUfKttLearuWrP9MDH5MBPbIqV92AaeXatLxBI9gBaebbnrfifHhDYfgasaacH8akY=wiFfYdH8Gipec8Eeeu0xXdbba9frFj0=OqFfea0dXdd9vqai=hGuQ8kuc9pgc9s8qqaq=dirpe0xb9q8qiLsFr0=vr0=vr0dc8meaabaqaciaacaGaaeqabaqabeGadaaakeaafaqaaeGabaaabaGaem4qamKaemyzauMaemOBa4Maem4CamNaem4Ba8MaemOCaiNaemyzauMaemizaqMaeeiiaaIaemizaqMaemyDauNaemOCaiNaemyAaKMaemOBa4Maem4zaCMaeeiiaaIaeiikaGIaeGimaaJaeyOeI0IaeGymaeJaeGOmaiJaeeiiaaIaemyBa0Maem4Ba8MaemOBa4MaemiDaqNaemiAaGMaeiykaKIaeiilaWIaeeiiaaIaemOCaiNaemyzauMaem4CamNaemyzauMaemyyaeMaemOCaiNaem4yamMaemiAaGMaeyypa0ZaaSaaaeaacqaIYaGmcqGHxdaTcqGGOaakcqaI0aancqaI5aqocqaIXaqmcqGHxdaTcqaI3aWncqaI4aaocqGHsislcqaIZaWmcqaI3aWncqaIYaGmcqGHxdaTcqaIXaqmcqaIWaamcqaIWaamcqGGPaqkaeaacqGGOaakcqaIXaqmcqaIWaamcqaIWaamcqGHRaWkcqaI3aWncqaI4aaocqGGPaqkaaGaeyypa0JaeGymaeJaeGOmaiJaeiOla4IaeG4mamJaeG4mamdabaGaem4qamKaemyzauMaemOBa4Maem4CamNaem4Ba8MaemOCaiNaemyzauMaemizaqMaeeiiaaIaemizaqMaemyDauNaemOCaiNaemyAaKMaemOBa4Maem4zaCMaeeiiaaIaeiikaGIaeGimaaJaeyOeI0IaeGymaeJaeGOmaiJaeeiiaaIaemyBa0Maem4Ba8MaemOBa4MaemiDaqNaemiAaGMaeiykaKIaeiilaWIaeeiiaaIaem4yamMaem4Ba8MaemOBa4MaemiDaqNaemOCaiNaem4Ba8MaemiBaWMaeyypa0ZaaSaaaeaacqaIYaGmcqGHxdaTcqGGOaakcqaI0aancqaI4aaocqaI1aqncqGHxdaTcqaI3aWncqaI1aqncqGHsislcqaIZaWmcqaI4aaocqaI1aqncqGHxdaTcqaIXaqmcqaIWaamcqaIWaamcqGGPaqkaeaacqGGOaakcqaIXaqmcqaIWaamcqaIWaamcqGHRaWkcqaI3aWncqaI1aqncqGGPaqkaaGaeyypa0JaeGymaeJaeGimaaJaeiOla4IaeGimaaJaeGimaadaaaaa@CD11@

approximately 12 and 10 patients were estimated to be censored on the research and control arm respectively. Note that in section 10, by estimating the minimum follow-up to be 14 months and using the censoring model, we failed to take accurate account of censoring in the 0–12 month period.

#### Step 4a. Estimate the HR and V for the current interval using the number of events and the numbers at risk during the current interval

The results from steps 1 and 2 can then be used to estimate the HR, *V *and *O-E *for the time interval using equations (21), (22) and (8), as in section 10.

#### Step 4b. Estimate the O-E and V and HR for the current interval using the numbers of events and the numbers at risk during the current interval

An alternative method estimates *E *and then *O-E *within in each interval:

Expected events during, research=(Events research+Events control)×At risk during, researchAt risk during, research+At risk during, control
 MathType@MTEF@5@5@+=feaafiart1ev1aaatCvAUfKttLearuWrP9MDH5MBPbIqV92AaeXatLxBI9gBaebbnrfifHhDYfgasaacH8akY=wiFfYdH8Gipec8Eeeu0xXdbba9frFj0=OqFfea0dXdd9vqai=hGuQ8kuc9pgc9s8qqaq=dirpe0xb9q8qiLsFr0=vr0=vr0dc8meaabaqaciaacaGaaeqabaqabeGadaaakeaacqWGfbqrcqWG4baEcqWGWbaCcqWGLbqzcqWGJbWycqWG0baDcqWGLbqzcqWGKbazcqqGGaaicqWGLbqzcqWG2bGDcqWGLbqzcqWGUbGBcqWG0baDcqWGZbWCcqqGGaaicqWGKbazcqWG1bqDcqWGYbGCcqWGPbqAcqWGUbGBcqWGNbWzcqGGSaalcqqGGaaicqWGYbGCcqWGLbqzcqWGZbWCcqWGLbqzcqWGHbqycqWGYbGCcqWGJbWycqWGObaAcqGH9aqpcqGGOaakcqWGfbqrcqWG2bGDcqWGLbqzcqWGUbGBcqWG0baDcqWGZbWCcqqGGaaicqWGYbGCcqWGLbqzcqWGZbWCcqWGLbqzcqWGHbqycqWGYbGCcqWGJbWycqWGObaAcqGHRaWkcqWGfbqrcqWG2bGDcqWGLbqzcqWGUbGBcqWG0baDcqWGZbWCcqqGGaaicqWGJbWycqWGVbWBcqWGUbGBcqWG0baDcqWGYbGCcqWGVbWBcqWGSbaBcqGGPaqkcqGHxdaTdaWcaaqaaiabdgeabjabdsha0jabbccaGiabdkhaYjabdMgaPjabdohaZjabdUgaRjabbccaGiabdsgaKjabdwha1jabdkhaYjabdMgaPjabd6gaUjabdEgaNjabcYcaSiabbccaGiabdkhaYjabdwgaLjabdohaZjabdwgaLjabdggaHjabdkhaYjabdogaJjabdIgaObqaaiabdgeabjabdsha0jabbccaGiabdkhaYjabdMgaPjabdohaZjabdUgaRjabbccaGiabdsgaKjabdwha1jabdkhaYjabdMgaPjabd6gaUjabdEgaNjabcYcaSiabbccaGiabdkhaYjabdwgaLjabdohaZjabdwgaLjabdggaHjabdkhaYjabdogaJjabdIgaOjabgUcaRiabdgeabjabdsha0jabbccaGiabdkhaYjabdMgaPjabdohaZjabdUgaRjabbccaGiabdsgaKjabdwha1jabdkhaYjabdMgaPjabd6gaUjabdEgaNjabcYcaSiabbccaGiabdogaJjabd+gaVjabd6gaUjabdsha0jabdkhaYjabd+gaVjabdYgaSbaaaaa@DD5D@

Using the data for the 0–12 month interval gives the *E *as:

Expected events during, research=(106.67+120.00)×484.83484.83+484.00=113.90
 MathType@MTEF@5@5@+=feaafiart1ev1aaatCvAUfKttLearuWrP9MDH5MBPbIqV92AaeXatLxBI9gBaebbnrfifHhDYfgasaacH8akY=wiFfYdH8Gipec8Eeeu0xXdbba9frFj0=OqFfea0dXdd9vqai=hGuQ8kuc9pgc9s8qqaq=dirpe0xb9q8qiLsFr0=vr0=vr0dc8meaabaqaciaacaGaaeqabaqabeGadaaakeaacqWGfbqrcqWG4baEcqWGWbaCcqWGLbqzcqWGJbWycqWG0baDcqWGLbqzcqWGKbazcqqGGaaicqWGLbqzcqWG2bGDcqWGLbqzcqWGUbGBcqWG0baDcqWGZbWCcqqGGaaicqWGKbazcqWG1bqDcqWGYbGCcqWGPbqAcqWGUbGBcqWGNbWzcqGGSaalcqqGGaaicqWGYbGCcqWGLbqzcqWGZbWCcqWGLbqzcqWGHbqycqWGYbGCcqWGJbWycqWGObaAcqGH9aqpcqGGOaakcqaIXaqmcqaIWaamcqaI2aGncqGGUaGlcqaI2aGncqaI3aWncqGHRaWkcqaIXaqmcqaIYaGmcqaIWaamcqGGUaGlcqaIWaamcqaIWaamcqGGPaqkcqGHxdaTdaWcaaqaaiabisda0iabiIda4iabisda0iabc6caUiabiIda4iabiodaZaqaaiabisda0iabiIda4iabisda0iabc6caUiabiIda4iabiodaZiabgUcaRiabisda0iabiIda4iabisda0iabc6caUiabicdaWiabicdaWaaacqGH9aqpcqaIXaqmcqaIXaqmcqaIZaWmcqGGUaGlcqaI5aqocqaIWaamaaa@7FD2@

And the *O-E*:

*O *- *E *=106.67 - 113.90 = -7.23

Either equation (12) or (13), described earlier can be use to estimate *V*. However, equation (13) is preferred if the randomisation ratio is not 1:1, or the numbers at risk during intervals are very different, e.g. because there is a big difference in effect between arms of the trial.

V=226.67×484.83×480(484.83+480)2=56.67
 MathType@MTEF@5@5@+=feaafiart1ev1aaatCvAUfKttLearuWrP9MDH5MBPbIqV92AaeXatLxBI9gBaebbnrfifHhDYfgasaacH8akY=wiFfYdH8Gipec8Eeeu0xXdbba9frFj0=OqFfea0dXdd9vqai=hGuQ8kuc9pgc9s8qqaq=dirpe0xb9q8qiLsFr0=vr0=vr0dc8meaabaqaciaacaGaaeqabaqabeGadaaakeaacqWGwbGvcqGH9aqpdaWcaaqaaiabikdaYiabikdaYiabiAda2iabc6caUiabiAda2iabiEda3iabgEna0kabisda0iabiIda4iabisda0iabc6caUiabiIda4iabiodaZiabgEna0kabisda0iabiIda4iabicdaWaqaaiabcIcaOiabisda0iabiIda4iabisda0iabc6caUiabiIda4iabiodaZiabgUcaRiabisda0iabiIda4iabicdaWiabcMcaPmaaCaaaleqabaGaeGOmaidaaaaakiabg2da9iabiwda1iabiAda2iabc6caUiabiAda2iabiEda3aaa@53B0@

Using equation (6) we can estimate a HR of 0.88 for the interval.

HR=exp⁡[−7.2356.67]=0.88
 MathType@MTEF@5@5@+=feaafiart1ev1aaatCvAUfKttLearuWrP9MDH5MBPbIqV92AaeXatLxBI9gBaebbnrfifHhDYfgasaacH8akY=wiFfYdH8Gipec8Eeeu0xXdbba9frFj0=OqFfea0dXdd9vqai=hGuQ8kuc9pgc9s8qqaq=dirpe0xb9q8qiLsFr0=vr0=vr0dc8meaabaqaciaacaGaaeqabaqabeGadaaakeaacqqGibascqqGsbGucqGH9aqpcyGGLbqzcqGG4baEcqGGWbaCdaWadaqaamaalaaabaGaeyOeI0IaeG4naCJaeiOla4IaeGOmaiJaeG4mamdabaGaeGynauJaeGOnayJaeiOla4IaeGOnayJaeG4naCdaaaGaay5waiaaw2faaiabg2da9iabicdaWiabc6caUiabiIda4iabiIda4aaa@447D@

#### Step 6, combining all time intervals

Taking all time intervals and censoring into account and using equation (1) as in section 10, gives a pooled HR of 0.88 and *V *of 119.80 (95%CI of 0.74–1.05).

### Interpreting the hazard ratio (HR)

Usually a HR calculated for a trial or a meta-analysis is interpreted as the relative risk of an event on the research arm compared to control. However, it can also be translated into an absolute difference in the proportion of patients who are event-free at a particular time point or for particular groups of patient, assuming proportional hazards:

exp [ln(proportion of patients event-free) × HR] - proportion event-free

Alternatively, it can be translated into an absolute difference in the median time event free, assuming exponential distributions, by first calculating the median time event free on the research arm:

Median time event free on controlHR
 MathType@MTEF@5@5@+=feaafiart1ev1aaatCvAUfKttLearuWrP9MDH5MBPbIqV92AaeXatLxBI9gBaebbnrfifHhDYfgasaacH8akY=wiFfYdH8Gipec8Eeeu0xXdbba9frFj0=OqFfea0dXdd9vqai=hGuQ8kuc9pgc9s8qqaq=dirpe0xb9q8qiLsFr0=vr0=vr0dc8meaabaqaciaacaGaaeqabaqabeGadaaakeaadaWcaaqaaiabb2eanjabbwgaLjabbsgaKjabbMgaPjabbggaHjabb6gaUjabbccaGiabbsha0jabbMgaPjabb2gaTjabbwgaLjabbccaGiabbwgaLjabbAha2jabbwgaLjabb6gaUjabbsha0jabbccaGiabbAgaMjabbkhaYjabbwgaLjabbwgaLjabbccaGiabb+gaVjabb6gaUjabbccaGiabbogaJjabb+gaVjabb6gaUjabbsha0jabbkhaYjabb+gaVjabbYgaSbqaaiabbIeaijabbkfasbaaaaa@58E3@

and then the difference between medians:

Median time event free on research - Median time event free on research

These measures require an estimate of the proportion of patients that are event-free in the control group or subgroup of interest and an estimate of the median time event-free in the control group, respectively. Such data may be obtained from a Kaplan-Meier curve of a representative trial or individual patient data meta-analysis, or even from epidemiological data. Alternatively, it may be possible to use 'typical' values from other literature.

Using the bladder cancer example, the HR of 0.85 and an estimated 2-year survival of 58% for patients on the control arm, gives an absolute improvement:

exp [ln(0.58) × 0.85] - 0.58 = 0.05

in survival of 5% at 2 years, taking it from 58% to 63%.

The median survival on control was estimated to be 37 months and so the median survival on the research arm is:

37.0/0.85 = 43.6

43.6 months, giving an absolute improvement in median survival:

43.6 - 37.0

of 6.6 months with the research treatment.

### Calculations spreadsheet

Some of the methods described are computationally more complex than others and performing all the calculations by hand for each and every trial can be laborious, lead to errors and require extra data checking. We have therefore developed spreadsheet in Microsoft Excel that carries out the calculations for all of the methods described. The user enters all the reported summary statistics and the spreadsheet estimates the HR, 95%CI, lnHR, *V*, and *O-E *by all possible methods. The user can also input data extracted from Kaplan-Meier curves and estimate censoring using the minimum and maximum follow-up or the reported numbers at risk, to obtain similar summary statistics. Graphical representations of the input data are produced for comparison with the published curves, to assist with data extraction or to highlight data entry errors. Results from all methods are provided in a single output screen, which facilitates comparison. The main features of the calculations spreadsheet are illustrated in Figure 2 and the spreadsheet itself is freely available to readers (see Additional file 1).

## Discussion

We have presented methods for calculating a HR and/or associated statistics from published time-to-event-analyses [1,2] into a practical, less statistical guide. A corresponding, easy-to-use calculations spreadsheet, to facilitate the computational aspects, is available from the authors. The resulting summary statistics can then be used in the meta-analysis procedures found in statistical and meta-analysis software.

There is a hierarchy in the methods described [1,2]. The direct methods make no assumptions and are preferable, followed by the various indirect methods based on reported statistics. The curve methods are likely to be the least reliable and it is not yet clear which method of adjusting for censoring is most reliable. If both curve methods are possible, the choice between the two may be a pragmatic one, depending on whether the minimum and maximum follow-up are reported or need to be estimated, and how many time points the number at risk are reported for and the event rate between those time points. The development of a hybrid of the two curve methods might optimise use of available data. Also, it is not clear how different schemes for dividing up the Kaplan Meier curves may impact on the resulting statistics. In fact, further research is required to assess how well all of the methods perform according to variations in, for example, trial size, levels of follow-up or event rates.

Although the methods provide a means of analysing time-to-event outcomes for individual trials, they cannot circumvent the other well-known problems of relying on only published data for systematic reviews and meta-analyses. For example, it may not be possible to include all relevant trials, either because trials are not published or because the trial report does not include the outcome of interest, situations which could lead to publication bias [11-13] or selective outcome reporting bias [14], respectively. Similarly, these methods cannot correct common problems with the original reported analyses, such as the exclusion of patients [15,16], analyses which are not by intention-to-treat [17] or analyses confined to particular patient subgroups, which may also lead to bias [16]. Furthermore, if the time-to-event outcome of interest is a long-term outcome, such as survival, then any HR estimation for an individual trial or meta-analysis will be limited by the extent of follow-up at the time that trials are reported. Such issues are relevant to all trials, systematic reviews and meta-analyses and so they should always be taken into account in interpreting results of these studies. Their relative impact is likely to vary between outcomes, trials, meta-analyses and healthcare areas and some may be addressed by obtaining further or updated information direct from trial investigators.

While the methods described previously [1,2] and elaborated here are not a substitute for the re-analysis IPD from all randomised patients, they offer the most appropriate way of analysing time-to-event outcomes, when IPD is not available or the approach is infeasible. Thus, whenever possible they should be used in preference to using a pooled OR or RR or a series of ORs or RRs at fixed time points. This should improve the quality of the analysis and subsequent interpretation of systematic reviews and meta-analyses that include time-to-event outcomes.

## Competing interests

The author(s) declare that they have no competing interests.

## Authors' contributions

This manuscript is based on workshops demonstrating these methods to systematic reviewers. JT helped develop the workshops and the spreadsheet to carry out the calculations, and drafted the manuscript. LS had the idea for the workshops, helped develop the initial methods paper and workshops and helped draft this manuscript. DG had the idea for the workshops, helped develop them and commented on the manuscript. SB helped test the spreadsheet and run the workshops and commented on the manuscript. MS developed the spreadsheet and commented on the manuscript. All authors read and approved the final manuscript.

## Appendix 1: Previously published formulae for generating hazard ratios from published time-to-event data [1,2]. The number in brackets link these to their descriptive equivalent in the text

1. Generating the O-E, V, HR and lnHR from reported summary statistics

For equations 1–16 and following the notation of Parmar *et al. *[1], for trial *i*:

*O*_*ri *_= observed number of events in the research group

*E*_*ri *_= logrank expected events in the research group

*O*_*ci *_= observed number of events in the control group

*E*_*ci *_= logrank expected events in the control group

*O*_*r *_- *E*_*r *_observed minus expected events in the research group

*O*_*i *_= total observed events (*O*_*ri *_+ *O*_*ci*_)

*V*_*ri *_= logrank variance

ln(HR_*i*_) = log HR

var[ln(HR_*i*_) = variance of the log hazard ratio

UPPCI_*i *_= Value for the upper end of the confidence interval

LOWCI_*i *_= Value for the lower end of the confidence interval

Φ^-1^(1-α_*i*_/2) = z score for the upper end of the confidence intreval

*R*_*ri *_= number randomised to the research group

*R*_*ci *_= number randomised to the control group

p_*i*_*= *reported two-sided p-value associated with the logrank or Mantel-Haenszel test (or Cox model)

Estimating a pooled lnHR from a series of trials

Estimating a pooled lnHR using the inverse variance method:

ln⁡(HR)=∑i=1Kln⁡(HRi)var⁡[ln⁡(HRi)]∑i=1K1var⁡[ln⁡(HRi)]
 MathType@MTEF@5@5@+=feaafiart1ev1aaatCvAUfKttLearuWrP9MDH5MBPbIqV92AaeXatLxBI9gBaebbnrfifHhDYfgasaacH8akY=wiFfYdH8Gipec8Eeeu0xXdbba9frFj0=OqFfea0dXdd9vqai=hGuQ8kuc9pgc9s8qqaq=dirpe0xb9q8qiLsFr0=vr0=vr0dc8meaabaqaciaacaGaaeqabaqabeGadaaakeaacyGGSbaBcqGGUbGBcqGGOaakcqqGibascqqGsbGucqGGPaqkcqGH9aqpdaWcaaqaamaaqahabaWaaSaaaeaacyGGSbaBcqGGUbGBcqGGOaakcqqGibascqqGsbGudaWgaaWcbaGaeeyAaKgabeaakiabcMcaPaqaaiGbcAha2jabcggaHjabckhaYjabcUfaBjGbcYgaSjabc6gaUjabcIcaOiabbIeaijabbkfasnaaBaaaleaacqqGPbqAaeqaaOGaeiykaKIaeiyxa0faaaWcbaGaemyAaKMaeyypa0JaeGymaedabaGaem4saSeaniabggHiLdaakeaadaaeWbqaamaalaaabaGaeGymaedabaGagiODayNaeiyyaeMaeiOCaiNaei4waSLagiiBaWMaeiOBa4MaeiikaGIaeeisaGKaeeOuai1aaSbaaSqaaiabbMgaPbqabaGccqGGPaqkcqGGDbqxaaaaleaacqWGPbqAcqGH9aqpcqaIXaqmaeaacqWGlbWsa0GaeyyeIuoaaaaaaa@692D@

Estimating the O-E, V, HR and lnHR from reported summary statistics

The reciprocal nature of the variance of the lnHR and the logrank variance:

var⁡(ln⁡(HRi))=1Vri
 MathType@MTEF@5@5@+=feaafiart1ev1aaatCvAUfKttLearuWrP9MDH5MBPbIqV92AaeXatLxBI9gBaebbnrfifHhDYfgasaacH8akY=wiFfYdH8Gipec8Eeeu0xXdbba9frFj0=OqFfea0dXdd9vqai=hGuQ8kuc9pgc9s8qqaq=dirpe0xb9q8qiLsFr0=vr0=vr0dc8meaabaqaciaacaGaaeqabaqabeGadaaakeaacyGG2bGDcqGGHbqycqGGYbGCcqGGOaakcyGGSbaBcqGGUbGBcqGGOaakcqqGibascqqGsbGudaWgaaWcbaGaemyAaKgabeaakiabcMcaPiabcMcaPiabg2da9maalaaabaGaeGymaedabaGaemOvay1aaSbaaSqaaiabbkhaYjabdMgaPbqabaaaaaaa@4102@

Vri=1var⁡(ln⁡(HRi))
 MathType@MTEF@5@5@+=feaafiart1ev1aaatCvAUfKttLearuWrP9MDH5MBPbIqV92AaeXatLxBI9gBaebbnrfifHhDYfgasaacH8akY=wiFfYdH8Gipec8Eeeu0xXdbba9frFj0=OqFfea0dXdd9vqai=hGuQ8kuc9pgc9s8qqaq=dirpe0xb9q8qiLsFr0=vr0=vr0dc8meaabaqaciaacaGaaeqabaqabeGadaaakeaacqWGwbGvdaWgaaWcbaGaeeOCaiNaemyAaKgabeaakiabg2da9maalaaabaGaeGymaedabaGagiODayNaeiyyaeMaeiOCaiNaeiikaGIagiiBaWMaeiOBa4MaeiikaGIaeeisaGKaeeOuai1aaSbaaSqaaiabdMgaPbqabaGccqGGPaqkcqGGPaqkaaaaaa@410C@

Directly estimating the lnHR and associated variance using the formal definition:

ln⁡(HRi)=ln⁡[Ori/EriOci/Eci]
 MathType@MTEF@5@5@+=feaafiart1ev1aaatCvAUfKttLearuWrP9MDH5MBPbIqV92AaeXatLxBI9gBaebbnrfifHhDYfgasaacH8akY=wiFfYdH8Gipec8Eeeu0xXdbba9frFj0=OqFfea0dXdd9vqai=hGuQ8kuc9pgc9s8qqaq=dirpe0xb9q8qiLsFr0=vr0=vr0dc8meaabaqaciaacaGaaeqabaqabeGadaaakeaacyGGSbaBcqGGUbGBcqGGOaakcqqGibascqqGsbGudaWgaaWcbaGaemyAaKgabeaakiabcMcaPiabg2da9iGbcYgaSjabc6gaUnaadmaabaWaaSaaaeaacqWGpbWtdaWgaaWcbaGaeeOCaiNaemyAaKgabeaakiabc+caViabdweafnaaBaaaleaacqqGYbGCcqWGPbqAaeqaaaGcbaGaem4ta80aaSbaaSqaaiabbogaJjabdMgaPbqabaGccqGGVaWlcqWGfbqrdaWgaaWcbaGaee4yamMaemyAaKgabeaaaaaakiaawUfacaGLDbaaaaa@4CB9@

Vri=1[(1/Eri)+(1/Eci)]
 MathType@MTEF@5@5@+=feaafiart1ev1aaatCvAUfKttLearuWrP9MDH5MBPbIqV92AaeXatLxBI9gBaebbnrfifHhDYfgasaacH8akY=wiFfYdH8Gipec8Eeeu0xXdbba9frFj0=OqFfea0dXdd9vqai=hGuQ8kuc9pgc9s8qqaq=dirpe0xb9q8qiLsFr0=vr0=vr0dc8meaabaqaciaacaGaaeqabaqabeGadaaakeaacqWGwbGvdaWgaaWcbaGaemOCaiNaemyAaKgabeaakiabg2da9maalaaabaGaeGymaedabaGaei4waSLaeiikaGIaeGymaeJaei4la8Iaemyrau0aaSbaaSqaaiabbkhaYjabdMgaPbqabaGccqGGPaqkcqGHRaWkcqGGOaakcqaIXaqmcqGGVaWlcqWGfbqrdaWgaaWcbaGaee4yamMaemyAaKgabeaakiabcMcaPiabc2faDbaaaaa@4557@

Direct estimation of the lnHR using the alternative definition:

ln⁡(HRi)=[Ori−EriVri]
 MathType@MTEF@5@5@+=feaafiart1ev1aaatCvAUfKttLearuWrP9MDH5MBPbIqV92AaeXatLxBI9gBaebbnrfifHhDYfgasaacH8akY=wiFfYdH8Gipec8Eeeu0xXdbba9frFj0=OqFfea0dXdd9vqai=hGuQ8kuc9pgc9s8qqaq=dirpe0xb9q8qiLsFr0=vr0=vr0dc8meaabaqaciaacaGaaeqabaqabeGadaaakeaacyGGSbaBcqGGUbGBcqGGOaakcqqGibascqqGsbGudaWgaaWcbaGaemyAaKgabeaakiabcMcaPiabg2da9maadmaabaWaaSaaaeaacqWGpbWtdaWgaaWcbaGaeeOCaiNaemyAaKgabeaakiabgkHiTiabdweafnaaBaaaleaacqqGYbGCcqWGPbqAaeqaaaGcbaGaemOvay1aaSbaaSqaaiabbkhaYjabdMgaPbqabaaaaaGccaGLBbGaayzxaaaaaa@454F@

Indirect estimation of the variance of the lnHR from the confidence interval:

var⁡(ln⁡HRi)=[UPPCIi−LOWCIi2Φ−1(1−αi/2)]2
 MathType@MTEF@5@5@+=feaafiart1ev1aaatCvAUfKttLearuWrP9MDH5MBPbIqV92AaeXatLxBI9gBaebbnrfifHhDYfgasaacH8akY=wiFfYdH8Gipec8Eeeu0xXdbba9frFj0=OqFfea0dXdd9vqai=hGuQ8kuc9pgc9s8qqaq=dirpe0xb9q8qiLsFr0=vr0=vr0dc8meaabaqaciaacaGaaeqabaqabeGadaaakeaacyGG2bGDcqGGHbqycqGGYbGCcqGGOaakcyGGSbaBcqGGUbGBcqqGibascqqGsbGudaWgaaWcbaGaemyAaKgabeaakiabcMcaPiabg2da9maadmaabaWaaSaaaeaacqqGvbqvcqqGqbaucqqGqbaucqqGdbWqcqqGjbqsdaWgaaWcbaGaemyAaKgabeaakiabgkHiTiabbYeamjabb+eapjabbEfaxjabboeadjabbMeajnaaBaaaleaacqWGPbqAaeqaaaGcbaGaeGOmaiJaeuOPdy0aaWbaaSqabeaacqGHsislcqaIXaqmaaGccqGGOaakcqaIXaqmcqGHsislcqaHXoqydaWgaaWcbaGaeeyAaKgabeaakiabc+caViabikdaYiabcMcaPaaaaiaawUfacaGLDbaadaahaaWcbeqaaiabikdaYaaaaaa@59B2@

Indirect estimation of the variance of the lnHR from the number of events:

*V*_*ri *_= *O*_*ri*_*O*_*ci*_/*O*_*i*_

*V*_*ri *_= *O*_*i*_/4

Indirect estimation of the variance of the lnHR from the number of events and the numbers randomised (analysed) on each arm:

Vri=OiRriRci(Rri+Rci)2
 MathType@MTEF@5@5@+=feaafiart1ev1aaatCvAUfKttLearuWrP9MDH5MBPbIqV92AaeXatLxBI9gBaebbnrfifHhDYfgasaacH8akY=wiFfYdH8Gipec8Eeeu0xXdbba9frFj0=OqFfea0dXdd9vqai=hGuQ8kuc9pgc9s8qqaq=dirpe0xb9q8qiLsFr0=vr0=vr0dc8meaabaqaciaacaGaaeqabaqabeGadaaakeaacqWGwbGvdaWgaaWcbaGaeeOCaiNaemyAaKgabeaakiabg2da9maalaaabaGaem4ta80aaSbaaSqaaiabdMgaPbqabaGccqWGsbGudaWgaaWcbaGaeeOCaiNaemyAaKgabeaakiabdkfasnaaBaaaleaacqqGJbWycqWGPbqAaeqaaaGcbaGaeiikaGIaemOuai1aaSbaaSqaaiabbkhaYjabdMgaPbqabaGccqGHRaWkcqWGsbGudaWgaaWcbaGaee4yamMaemyAaKgabeaakiabcMcaPmaaCaaaleqabaGaeGOmaidaaaaaaaa@48C6@

Indirect estimation of the observed minus expected events from the observed events and the p-value:

(Ori−Eri)=OriOciOi×Φ−1(1−pi2)
 MathType@MTEF@5@5@+=feaafiart1ev1aaatCvAUfKttLearuWrP9MDH5MBPbIqV92AaeXatLxBI9gBaebbnrfifHhDYfgasaacH8akY=wiFfYdH8Gipec8Eeeu0xXdbba9frFj0=OqFfea0dXdd9vqai=hGuQ8kuc9pgc9s8qqaq=dirpe0xb9q8qiLsFr0=vr0=vr0dc8meaabaqaciaacaGaaeqabaqabeGadaaakeaacqGGOaakcqWGpbWtdaWgaaWcbaGaeeOCaiNaemyAaKgabeaakiabgkHiTiabdweafnaaBaaaleaacqqGYbGCcqWGPbqAaeqaaOGaeiykaKIaeyypa0ZaaOaaaeaadaWcaaqaaiabd+eapnaaBaaaleaacqqGYbGCcqWGPbqAaeqaaOGaem4ta80aaSbaaSqaaiabbogaJjabdMgaPbqabaaakeaacqWGpbWtdaWgaaWcbaGaemyAaKgabeaaaaaabeaakiabgEna0kabfA6agnaaCaaaleqabaGaeyOeI0IaeGymaedaaOGaeiikaGIaeGymaeJaeyOeI0YaaSaaaeaacqWGWbaCdaWgaaWcbaGaemyAaKgabeaaaOqaaiabikdaYaaacqGGPaqkaaa@50B3@

(Ori−Eri)=1/2×Oi×Φ−1(1−pi2)
 MathType@MTEF@5@5@+=feaafiart1ev1aaatCvAUfKttLearuWrP9MDH5MBPbIqV92AaeXatLxBI9gBaebbnrfifHhDYfgasaacH8akY=wiFfYdH8Gipec8Eeeu0xXdbba9frFj0=OqFfea0dXdd9vqai=hGuQ8kuc9pgc9s8qqaq=dirpe0xb9q8qiLsFr0=vr0=vr0dc8meaabaqaciaacaGaaeqabaqabeGadaaakeaacqGGOaakcqWGpbWtdaWgaaWcbaGaeeOCaiNaemyAaKgabeaakiabgkHiTiabdweafnaaBaaaleaacqqGYbGCcqWGPbqAaeqaaOGaeiykaKIaeyypa0JaeGymaeJaei4la8IaeGOmaiJaey41aq7aaOaaaeaacqWGpbWtdaWgaaWcbaGaemyAaKgabeaaaeqaaOGaey41aqRaeuOPdy0aaWbaaSqabeaacqGHsislcqaIXaqmaaGccqGGOaakcqaIXaqmcqGHsisldaWcaaqaaiabdchaWnaaBaaaleaacqWGPbqAaeqaaaGcbaGaeGOmaidaaiabcMcaPaaa@4D5A@

Indirect estimation of the observed minus expected events from the observed events, the p-value and the numbers randomised (analysed) on each arm:

(Ori−Eri)=(OiRriRci(Rri+Rci)×Φ−1(1−pi2)
 MathType@MTEF@5@5@+=feaafiart1ev1aaatCvAUfKttLearuWrP9MDH5MBPbIqV92AaeXatLxBI9gBaebbnrfifHhDYfgasaacH8akY=wiFfYdH8Gipec8Eeeu0xXdbba9frFj0=OqFfea0dXdd9vqai=hGuQ8kuc9pgc9s8qqaq=dirpe0xb9q8qiLsFr0=vr0=vr0dc8meaabaqaciaacaGaaeqabaqabeGadaaakeaacqGGOaakcqWGpbWtdaWgaaWcbaGaeeOCaiNaemyAaKgabeaakiabgkHiTiabdweafnaaBaaaleaacqqGYbGCcqWGPbqAaeqaaOGaeiykaKIaeyypa0ZaaSaaaeaadaGcaaqaaiabcIcaOiabd+eapnaaBaaaleaacqWGPbqAaeqaaOGaemOuai1aaSbaaSqaaiabbkhaYjabdMgaPbqabaGccqWGsbGudaWgaaWcbaGaee4yamMaemyAaKgabeaaaeqaaaGcbaGaeiikaGIaemOuai1aaSbaaSqaaiabbkhaYjabdMgaPbqabaGccqGHRaWkcqWGsbGudaWgaaWcbaGaee4yamMaemyAaKgabeaakiabcMcaPaaacqGHxdaTcqqHMoGrdaahaaWcbeqaaiabgkHiTiabigdaXaaakiabcIcaOiabigdaXiabgkHiTmaalaaabaGaemiCaa3aaSbaaSqaaiabdMgaPbqabaaakeaacqaIYaGmaaGaeiykaKcaaa@5C5F@

2. Generating the HR and V from published Kaplan-Meier curves and follow-up

For equations 17–22, and following the notation of Parmar *et al. *[1], for trial *i *and *T *non-overlapping time points (*t *= 1, ...,*T*) :

*t *= whole time interval (*t - *1, *t*)

*t*_*s *_= start of the time interval (*t - *1, *t*)

*t*_*e *_= end of the time interval (*t - *1, *t*)

*R*_*ri*_(*t*) = effective number of patients at risk on the research arm during time interval (*t - *1, *t*)

*R*_*ri*_(*t - *1) = effective number of patients at risk on the research arm during time interval *(t - 2*,*t - *1)

*D*_*ri*_(*t*) = effective number of events on the research arm during time interval (*t - *1, *t*)

*D*_*ci*_(*t*) = effective number of events on the control arm during time interval (*t - *1, *t*)

*D*_*ri*_(*t - *1) = effective number of events on the research arm during time interval *(t - 2*,*t - *1)

*C*_*ri*_(*t*) = effective number of patients censored on the research arm during time interval (*t - *1, *t*)

*C*_*ci*_(*t*) = effective number of patients censored on the control arm during time interval (*t - *1, *t*)

*C*_*ri*_(*t - 1*) = effective number of patients censored on the research arm during time interval (*t - *2, *t - 1)*

S_*ri*_(*t*_*s*_) = event-free probability on the research arm at the start of time interval (*t - *1, *t*)

S_*ri*_(*t*_*e*_) = event-free probability on the research arm at the end of time interval (*t - *1, *t*)

*F*_*min *_= minimum follow-up

*F*_*max *_= maximum follow-up

Estimation of the numbers event-free at the start of a time interval:

*R*_*ri*_(*t*_*s*_) = *R*_*ri*_(*t *- 1)- *D*_*ri*_(*t *- 1) - *C*_*ri*_(*t *- 1)

Estimation of the numbers censored during a time interval

if ts≥Fmin⁡ and Fmin≤te≤Fmax⁡Cri(t)=Rri(ts){12(te−ts)(Fmax⁡−ts)} (assuming censoring at constant rate)
 MathType@MTEF@5@5@+=feaafiart1ev1aaatCvAUfKttLearuWrP9MDH5MBPbIqV92AaeXatLxBI9gBaebbnrfifHhDYfgasaacH8akY=wiFfYdH8Gipec8Eeeu0xXdbba9frFj0=OqFfea0dXdd9vqai=hGuQ8kuc9pgc9s8qqaq=dirpe0xb9q8qiLsFr0=vr0=vr0dc8meaabaqaciaacaGaaeqabaqabeGadaaakeaafaqaaeGabaaabaGaeeyAaKMaeeOzayMaeeiiaaIaemiDaq3aaSbaaSqaaiabdohaZbqabaGccqGHLjYScqWGgbGrdaWgaaWcbaGagiyBa0MaeiyAaKMaeiOBa4gabeaakiabbccaGiabbggaHjabb6gaUjabbsgaKjabbccaGiabdAeagnaaBaaaleaacqqGTbqBcqqGPbqAcqqGUbGBaeqaaOGaeyizImQaemiDaq3aaSbaaSqaaiabdwgaLbqabaGccqGHKjYOcqWGgbGrdaWgaaWcbaGagiyBa0MaeiyyaeMaeiiEaGhabeaaaOqaaiabdoeadnaaBaaaleaacqqGYbGCcqWGPbqAaeqaaOGaeiikaGIaemiDaqNaeiykaKIaeyypa0JaemOuai1aaSbaaSqaaiabbkhaYjabdMgaPbqabaGccqGGOaakcqWG0baDdaWgaaWcbaGaee4CamhabeaakiabcMcaPmaacmqabaWaaSaaaeaacqaIXaqmaeaacqaIYaGmaaWaaSaaaeaacqGGOaakcqWG0baDdaWgaaWcbaGaeeyzaugabeaakiabgkHiTiabdsha0naaBaaaleaacqqGZbWCaeqaaOGaeiykaKcabaGaeiikaGIaemOray0aaSbaaSqaaiGbc2gaTjabcggaHjabcIha4bqabaGccqGHsislcqWG0baDdaWgaaWcbaGaee4CamhabeaakiabcMcaPaaaaiaawUhacaGL9baacqqGGaaicqGGOaakcqqGHbqycqqGZbWCcqqGZbWCcqqG1bqDcqqGTbqBcqqGPbqAcqqGUbGBcqqGNbWzcqqGGaaicqqGJbWycqqGLbqzcqqGUbGBcqqGZbWCcqqGVbWBcqqGYbGCcqqGPbqAcqqGUbGBcqqGNbWzcqqGGaaicqqGHbqycqqG0baDcqqGGaaicqqGJbWycqqGVbWBcqqGUbGBcqqGZbWCcqqG0baDcqqGHbqycqqGUbGBcqqG0baDcqqGGaaicqqGYbGCcqqGHbqycqqG0baDcqqGLbqzcqGGPaqkaaaaaa@AA82@

Estimation of the numbers at risk during a time interval, adjusted for censoring

*R*_*ri*_(*t*) = *R*_*ri*_(*t*_*s*_)- *C*_*ri*_(*t*)

Estimation of the number of events during a time interval

Dri(t)=[Rri(t)×(Sri(ts)−Sri(te)Sri(ts))]
 MathType@MTEF@5@5@+=feaafiart1ev1aaatCvAUfKttLearuWrP9MDH5MBPbIqV92AaeXatLxBI9gBaebbnrfifHhDYfgasaacH8akY=wiFfYdH8Gipec8Eeeu0xXdbba9frFj0=OqFfea0dXdd9vqai=hGuQ8kuc9pgc9s8qqaq=dirpe0xb9q8qiLsFr0=vr0=vr0dc8meaabaqaciaacaGaaeqabaqabeGadaaakeaacqWGebardaWgaaWcbaGaeeOCaiNaemyAaKgabeaakiabcIcaOiabdsha0jabcMcaPiabg2da9maadmaabaGaemOuai1aaSbaaSqaaiabbkhaYjabdMgaPbqabaGccqGGOaakcqWG0baDcqGGPaqkcqGHxdaTdaqadaqaamaalaaabaGaem4uam1aaSbaaSqaaiabbkhaYjabdMgaPbqabaGccqGGOaakcqWG0baDdaWgaaWcbaGaee4CamhabeaakiabcMcaPiabgkHiTiabdofatnaaBaaaleaacqqGYbGCcqWGPbqAaeqaaOGaeiikaGIaemiDaq3aaSbaaSqaaiabbwgaLbqabaGccqGGPaqkaeaacqWGtbWudaWgaaWcbaGaeeOCaiNaemyAaKgabeaakiabcIcaOiabdsha0naaBaaaleaacqqGZbWCaeqaaOGaeiykaKcaaaGaayjkaiaawMcaaaGaay5waiaaw2faaaaa@5D74@

Note that equations 17–20 are also are used for the control arm.

Estimation of the HR and *V *for a time interval from a Kaplan-Meier curve

ln⁡(HRi(t))=ln⁡(Dri(t)/Rri(t)Dci(t)/Rci(t))
 MathType@MTEF@5@5@+=feaafiart1ev1aaatCvAUfKttLearuWrP9MDH5MBPbIqV92AaeXatLxBI9gBaebbnrfifHhDYfgasaacH8akY=wiFfYdH8Gipec8Eeeu0xXdbba9frFj0=OqFfea0dXdd9vqai=hGuQ8kuc9pgc9s8qqaq=dirpe0xb9q8qiLsFr0=vr0=vr0dc8meaabaqaciaacaGaaeqabaqabeGadaaakeaacyGGSbaBcqGGUbGBcqGGOaakcqqGibascqqGsbGudaWgaaWcbaGaeeyAaKgabeaakiabcIcaOiabdsha0jabcMcaPiabcMcaPiabg2da9iGbcYgaSjabc6gaUnaabmaabaWaaSaaaeaacqWGebardaWgaaWcbaGaeeOCaiNaemyAaKgabeaakiabcIcaOiabdsha0jabcMcaPiabc+caViabdkfasnaaBaaaleaacqqGYbGCcqWGPbqAaeqaaOGaeiikaGIaemiDaqNaeiykaKcabaGaemiraq0aaSbaaSqaaiabbogaJjabdMgaPbqabaGccqGGOaakcqWG0baDcqGGPaqkcqGGVaWlcqWGsbGudaWgaaWcbaGaee4yamMaemyAaKgabeaakiabcIcaOiabdsha0jabcMcaPaaaaiaawIcacaGLPaaaaaa@5C05@

var⁡[ln⁡(HRi(t))]=1Dri(t)−1Rri(t)+1Dci(t)−1Rci(t)
 MathType@MTEF@5@5@+=feaafiart1ev1aaatCvAUfKttLearuWrP9MDH5MBPbIqV92AaeXatLxBI9gBaebbnrfifHhDYfgasaacH8akY=wiFfYdH8Gipec8Eeeu0xXdbba9frFj0=OqFfea0dXdd9vqai=hGuQ8kuc9pgc9s8qqaq=dirpe0xb9q8qiLsFr0=vr0=vr0dc8meaabaqaciaacaGaaeqabaqabeGadaaakeaacyGG2bGDcqGGHbqycqGGYbGCcqGGBbWwcyGGSbaBcqGGUbGBcqGGOaakcqqGibascqqGsbGudaWgaaWcbaGaeeyAaKgabeaakiabcIcaOiabdsha0jabcMcaPiabcMcaPiabc2faDjabg2da9maalaaabaGaeGymaedabaGaemiraq0aaSbaaSqaaiabbkhaYjabdMgaPbqabaGccqGGOaakcqWG0baDcqGGPaqkaaGaeyOeI0YaaSaaaeaacqaIXaqmaeaacqWGsbGudaWgaaWcbaGaeeOCaiNaemyAaKgabeaakiabcIcaOiabdsha0jabcMcaPaaacqGHRaWkdaWcaaqaaiabigdaXaqaaiabdseaenaaBaaaleaacqqGJbWycqWGPbqAaeqaaOGaeiikaGIaemiDaqNaeiykaKcaaiabgkHiTmaalaaabaGaeGymaedabaGaemOuai1aaSbaaSqaaiabbogaJjabdMgaPbqabaGccqGGOaakcqWG0baDcqGGPaqkaaaaaa@6342@

3. Generating the HR and V from published Kaplan-Meier curves and the numbers at risk

For equations 23–26, and following the notation of [2], for time interval *i*:

*j *= treatment group (where 1 = the control arm and 2= the research arm)

*t*_*i*-1 _= time at the start of the current interval

*t*_*i*-1_= time at the start of the prior interval

*n*_*j*,*i *_= number at risk at end of interval [*t*_*i*-1,_*t*_*i*_) in group *j*

*n*_*j*,*i*-1 _= number at risk at start of interval [*t*_*i*-1,_*t*_*i*_) in group *j*

n*_j,*i *_= number at risk during interval [*t*_*i*-1,_*t*_*i*_) in group *j*

d*_j,*i *_= number of events during interval [*t*_*i*-1,_*t*_*i*_) in group *j*

c*_j,*i *_= number censored during interval [*t*_*i*-1,_*t*_*i*_) in group *j*

s*_j,*i *_= event-free probability at end of interval [*t*_*i*-1,_*t*_*i*_) in group *j*

s*_j,*i*-1 _= event-free probability at start of interval [*t*_*i*-1,_*t*_*i*_) in group *j*

e*_j,*i *_= logrank expected events during interval [*t*_*i*-1,_*t*_*i*_) in group j = 2 (the research arm)

Estimation of the numbers at risk during a time interval from a Kaplan-Meier curve

nj,i∗=(nj,i−1+nj,i)sj,i−1∗(sj,i−1∗+sj,i∗)
 MathType@MTEF@5@5@+=feaafiart1ev1aaatCvAUfKttLearuWrP9MDH5MBPbIqV92AaeXatLxBI9gBaebbnrfifHhDYfgasaacH8akY=wiFfYdH8Gipec8Eeeu0xXdbba9frFj0=OqFfea0dXdd9vqai=hGuQ8kuc9pgc9s8qqaq=dirpe0xb9q8qiLsFr0=vr0=vr0dc8meaabaqaciaacaGaaeqabaqabeGadaaakeaacqWGUbGBdaqhaaWcbaGaemOAaOMaeiilaWIaemyAaKgabaGaey4fIOcaaOGaeyypa0ZaaSaaaeaacqGGOaakcqWGUbGBdaWgaaWcbaGaemOAaOMaeiilaWIaemyAaKMaeyOeI0IaeGymaedabeaakiabgUcaRiabd6gaUnaaBaaaleaacqWGQbGAcqGGSaalcqWGPbqAaeqaaOGaeiykaKIaem4Cam3aa0baaSqaaiabdQgaQjabcYcaSiabdMgaPjabgkHiTiabigdaXaqaaiabgEHiQaaaaOqaaiabcIcaOiabdohaZnaaDaaaleaacqWGQbGAcqGGSaalcqWGPbqAcqGHsislcqaIXaqmaeaacqGHxiIkaaGccqGHRaWkcqWGZbWCdaqhaaWcbaGaemOAaOMaeiilaWIaemyAaKgabaGaey4fIOcaaOGaeiykaKcaaaaa@5B91@

Estimation of the number of events during a time interval from a Kaplan-Meier curve

dj,i∗=(nj,i−1+nj,i)(sj,i−1∗−sj,i∗)(sj,i−1∗+sj,i∗)
 MathType@MTEF@5@5@+=feaafiart1ev1aaatCvAUfKttLearuWrP9MDH5MBPbIqV92AaeXatLxBI9gBaebbnrfifHhDYfgasaacH8akY=wiFfYdH8Gipec8Eeeu0xXdbba9frFj0=OqFfea0dXdd9vqai=hGuQ8kuc9pgc9s8qqaq=dirpe0xb9q8qiLsFr0=vr0=vr0dc8meaabaqaciaacaGaaeqabaqabeGadaaakeaacqWGKbazdaqhaaWcbaGaemOAaOMaeiilaWIaemyAaKgabaGaey4fIOcaaOGaeyypa0ZaaSaaaeaacqGGOaakcqWGUbGBdaWgaaWcbaGaemOAaOMaeiilaWIaemyAaKMaeyOeI0IaeGymaedabeaakiabgUcaRiabd6gaUnaaBaaaleaacqWGQbGAcqGGSaalcqWGPbqAaeqaaOGaeiykaKIaeiikaGIaem4Cam3aa0baaSqaaiabdQgaQjabcYcaSiabdMgaPjabgkHiTiabigdaXaqaaiabgEHiQaaakiabgkHiTiabdohaZnaaDaaaleaacqWGQbGAcqGGSaalcqWGPbqAaeaacqGHxiIkaaGccqGGPaqkaeaacqGGOaakcqWGZbWCdaqhaaWcbaGaemOAaOMaeiilaWIaemyAaKMaeyOeI0IaeGymaedabaGaey4fIOcaaOGaey4kaSIaem4Cam3aa0baaSqaaiabdQgaQjabcYcaSiabdMgaPbqaaiabgEHiQaaakiabcMcaPaaaaaa@6449@

Estimation of the numbers censored during a time interval from a Kaplan-Meier curve

cj,i∗=2(nj,i−1sj,i∗−nj,isj,i−1∗)(sj,i−1∗+sj,i∗)
 MathType@MTEF@5@5@+=feaafiart1ev1aaatCvAUfKttLearuWrP9MDH5MBPbIqV92AaeXatLxBI9gBaebbnrfifHhDYfgasaacH8akY=wiFfYdH8Gipec8Eeeu0xXdbba9frFj0=OqFfea0dXdd9vqai=hGuQ8kuc9pgc9s8qqaq=dirpe0xb9q8qiLsFr0=vr0=vr0dc8meaabaqaciaacaGaaeqabaqabeGadaaakeaacqWGJbWydaqhaaWcbaGaemOAaOMaeiilaWIaemyAaKgabaGaey4fIOcaaOGaeyypa0ZaaSaaaeaacqaIYaGmcqGGOaakcqWGUbGBdaWgaaWcbaGaemOAaOMaeiilaWIaemyAaKMaeyOeI0IaeGymaedabeaakiabdohaZnaaDaaaleaacqWGQbGAcqGGSaalcqWGPbqAaeaacqGHxiIkaaGccqGHsislcqWGUbGBdaWgaaWcbaGaemOAaOMaeiilaWIaemyAaKgabeaakiabdohaZnaaDaaaleaacqWGQbGAcqGGSaalcqWGPbqAcqGHsislcqaIXaqmaeaacqGHxiIkaaGccqGGPaqkaeaacqGGOaakcqWGZbWCdaqhaaWcbaGaemOAaOMaeiilaWIaemyAaKMaeyOeI0IaeGymaedabaGaey4fIOcaaOGaey4kaSIaem4Cam3aa0baaSqaaiabdQgaQjabcYcaSiabdMgaPbqaaiabgEHiQaaakiabcMcaPaaaaaa@62A5@

Estimation of the number of logrank expected events during a time interval from a Kaplan-Meier curve

e2,i∗=(d2,i∗+d1,i∗)∗n2,i∗n2,i∗+n1,i∗
 MathType@MTEF@5@5@+=feaafiart1ev1aaatCvAUfKttLearuWrP9MDH5MBPbIqV92AaeXatLxBI9gBaebbnrfifHhDYfgasaacH8akY=wiFfYdH8Gipec8Eeeu0xXdbba9frFj0=OqFfea0dXdd9vqai=hGuQ8kuc9pgc9s8qqaq=dirpe0xb9q8qiLsFr0=vr0=vr0dc8meaabaqaciaacaGaaeqabaqabeGadaaakeaacqWGLbqzdaqhaaWcbaGaeGOmaiJaeiilaWIaemyAaKgabaGaey4fIOcaaOGaeyypa0JaeiikaGIaemizaq2aa0baaSqaaiabikdaYiabcYcaSiabdMgaPbqaaiabgEHiQaaakiabgUcaRiabdsgaKnaaDaaaleaacqaIXaqmcqGGSaalcqWGPbqAaeaacqGHxiIkaaGccqGGPaqkcqGHxiIkdaWcaaqaaiabd6gaUnaaDaaaleaacqaIYaGmcqGGSaalcqWGPbqAaeaacqGHxiIkaaaakeaacqWGUbGBdaqhaaWcbaGaeGOmaiJaeiilaWIaemyAaKgabaGaey4fIOcaaOGaey4kaSIaemOBa42aa0baaSqaaiabigdaXiabcYcaSiabdMgaPbqaaiabgEHiQaaaaaaaaa@542F@

## Appendix 2: Estimating or educated 'guesstimating' minimum and maximum follow-up

When the minimum and maximum follow-up are not explicitly reported, it may be possible to estimate them for a particular trial, provided that some indicators of extent of follow-up are provided. In descending order of preference, the following are some strategies that we have employed to estimate the minimum and maximum follow-up:

### For minimum follow-up, if the trial report presents

1. Censoring tick marks on Kaplan-Meier curve

 Assume first tick mark indicates the point of minimum follow-up

2. Median follow-up and accrual period

 Assume minimum follow-up = median follow-up minus half the accrual period

3. Date of analysis and accrual period, could assume

 Assume minimum follow-up = date of analysis minus final date of accrual

4. Date of submission and accrual period

 Assume estimated date of analysis = date of submission minus 6 months

 Assume minimum follow-up = estimated date of analysis minus final date of accrual

### For maximum follow-up, if the trial report presents

1. Censoring tick marks on Kaplan-Meier curve

 Assume last tick mark indicates the point of maximum follow-up

2. Median follow-up and accrual period

 Assume maximum follow-up = median follow-up plus half the accrual period

3. Date of analysis and accrual period, could assume

 Assume maximum follow-up = date of analysis minus first date of accrual

4. Date of submission and accrual period, could assume

 Assume estimated date of analysis = date of submission minus 6 months

Assume maximum follow-up = estimated date of analysis minus first date of accrual

## Supplementary Material

Additional file 1 HR calculations spreadsheet. Spreadsheet to facilitate the estimation of hazard ratios from published summary statistics or data extracted from Kaplan-Meier curves. Click here for file
